# Synthesis of Altissimacoumarin D and Other Prenylated Coumarins and Their Ability to Reverse the Multidrug Resistance Phenotype in *Candida albicans*

**DOI:** 10.3390/jof9070758

**Published:** 2023-07-18

**Authors:** Anna Claudia Silva, Daniel Clemente de Moraes, Denilson Costa do Carmo, Giselle Cristina Casaes Gomes, A. Ganesan, Rosangela Sabbatini Capella Lopes, Antonio Ferreira-Pereira, Cláudio Cerqueira Lopes

**Affiliations:** 1 Departamento de Química Analítica, Instituto de Química, Universidade Federal do Rio de Janeiro, Centro de Tecnologia, Bloco A, 508, Rio de Janeiro 21949-900, Brazil; 2 Departamento de Microbiologia Geral, Instituto de Microbiologia Paulo de Góes, Universidade Federal do Rio de Janeiro, Centro de Ciências da Saúde, Bloco I, 44, Rio de Janeiro 21941-902, Brazil; 3 School of Pharmacy, University of East Anglia, Norwich Research Park, Norwich NR4 7TJ, UK

**Keywords:** altissimacoumarin D, *Candida* spp., coumarins, efflux transporters, isofraxidin, multidrug resistance

## Abstract

Azoles are the main antifungal agents employed in clinical practice to treat invasive candidiasis. Nonetheless, their efficacy is limited by fungal resistance mechanisms, mainly the overexpression of efflux pumps. Consequently, candidiasis has a worrisome death rate of 75%. One potential strategy to overcome efflux-mediated resistance is to inhibit this process. *Ailanthus altissima* is a Chinese tree that produces several active substances, including altissimacoumarin D. Due to the low yield of its extraction and the need to search for new drugs to treat candidiasis, this study aimed to synthesize altissimacoumarin D and its analogues, as well as evaluating their ability to reverse the resistance phenotype of *Candida albicans*. Coumarin isofraxidin was prepared via total synthesis through a solvent-free Knoevenagel condensation as the key step. Isofraxidin and other commercially available coumarins were alkylated with prenyl or geranyl groups to yield the natural product altissimacoumarin D and seven analogues. The antifungal activity of the coumarins and their ability to reverse the fungal resistance phenotype were assessed using microbroth methodologies. Toxicity was evaluated using erythrocytes and an in silico prediction. All compounds improved the antifungal activity of fluconazole by inhibiting efflux pumps, and ACS47 and ACS50 were the most active. None of the coumarins were toxic to erythrocytes. In silico predictions indicate that ACS47 and ACS50 may be safe for human use. ACS47 and ACS50 are promising candidates when used as adjuvants in the antifungal therapy against *C. albicans*-resistant strains.

## 1. Introduction

Fungi belonging to the *Candida* genus can be found within the human microbiome, at sites such as skin and urinary tract [1]. Nonetheless, these microorganisms may invade inner organs and cause life-threatening infections, mainly in immunocompromised patients. In this context, *Candida* spp. is the most significant fungal pathogen in humans, and its infection has mortality rates of 46–75% [2].

Currently, treating invasive fungal infections relies on a limited number of antifungal agents—azoles, polyenes and echinocandins—and the increasing incidence of resistance to them jeopardizes the success of therapy [3]. Azole drugs, such as fluconazole, itraconazole and ketoconazole, are first-line treatments and are subject to several mechanisms of resistance by *Candida* spp. [4].

The most important treatment involves the overexpression of efflux pumps within the plasma membrane [5]. These transporters may belong to the ATP-Binding Cassette (ABC) superfamily, which uses ATP hydrolysis as energy source to extrude drugs from the cytoplasm to the extracellular milieu [6], or to the Major Facilitator Superfamily (MFS), which uses proton gradient to transport the drugs [7] and confers a multidrug resistance (MDR) phenotype. Overcoming efflux-pump-mediated resistance is thereby an important strategy for combating fungal resistance to current drugs [8].

*Ailanthus altissima* is a Chinese native tree with a history of use in traditional herbal medicines for a variety of ailments that range from vaginal infections to epilepsy. Several compounds can be extracted and isolated from the plant, including approximately one hundred coumarins. These substances mostly show antitumor and deacetylating activity. Nonetheless, they are not the most studied group of compounds found in *A. altissima* and require further investigation [9]. Coumarins, and especially prenylated coumarins, have already been studied and have antibacterial [10,11], antifungal [12], antileishmanial [13], anticancer [14], anti-inflammatory and antiviral [15] activities.

**Figure 1:**
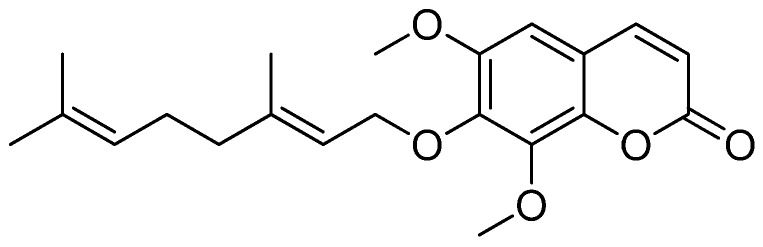
Structure of altissimacoumarin D (ACS51).

In 2012, a group of coumarins was isolated for the first time from *A. altissima,* and their deacetylating activity was demonstrated. However, no other studies have been performed since then, which was probably a consequence of the difficulty of obtaining such compounds, considering that 3 kg of the tree bark provides 6.5 to 22 mg of the coumarins [16]. In a previous study by our group, we were able to perform the first total synthesis of altissimacoumarin D (Figure 1), the most active compound isolated from *A. altissima* by Dao et al. [17].

In this study, we aimed to improve the total synthesis of altissimacoumarin D by decreasing the number of steps and toxic reagents used, avoiding high temperatures (e.g., ethylene glycol reflux) and the use of anhydrous solvents. Analogues were also synthesized, and we demonstrated that these compounds have the ability to reverse the MDR phenotype in *Candida albicans*.

## 2. Experimental Section

### 2.1. General Experiment Procedure

All reagents were obtained commercially (Sigma-Aldrich Co., St. Louis, MO, USA) and used without further purification, except fluconazole which was obtained commercially from the university pharmacy (UFJF, Juiz de Fora—MG, Brazil). Analytical thin-layer chromatography was carried out on 0.25 mm silica gel 60 F254 plates, and compounds were visualized using UV irradiation or staining with 2,4-dinitrophenylhydrazine. Nuclear magnetic resonance (NMR) spectra were obtained on a Bruker Avance-III spectrometer (Bruker Corporation, Billerica, MA, USA) (Figures S1–S16). The splitting patterns are reported as s (singlet), d (doublet), t (triplet), q (quartet) and m (multiplet). Fluconazole stock solutions were prepared in distilled water, sterilized by filtration (0.22 μm), and maintained at −20 °C.

### 2.2. Synthetic Procedures

#### 2.2.1. 3,5-Dibromo-2,4-dihydroxybenzaldehyde

This compound was prepared as previously described [17]. ¹H NMR (400 MHz, DMSO-*d*_6_) δ 7.96 (s, 1H), 9.78 (s, 1H); ¹³C NMR (100 MHz, DMSO-*d*_6_) δ 100.1, 101.9, 116.3, 135.7, 158.1, 158.7, 193.5.

#### 2.2.2. 2,4-Dihydroxy-3,5-dimethoxybenzaldehyde

This compound was prepared as previously described [17]. ¹H NMR (400 MHz, CDCl_3_) δ 3.98 (s, 6H), 7.28 (s, 1H), 8.0 (s, 1H); ¹³C NMR (100 MHz, CDCl_3_) δ 56.6, 60.9, 109.0, 112.9, 134.6, 141.3, 147.1, 151.7, 194.6.

#### 2.2.3. 7-Hydroxy-6,8-dimethoxy-2H-chromen-2-one (isofraxidin)

2,4-dihydroxy-3,5-dimethoxybenzaldehyde (1 eq), Meldrum’s acid (1.2 eq) and ammonium carbonate (0.2 eq) were placed in a sealed reaction tube and stirred at 75 °C for 2 h. The reaction was monitored using thin-layer chromatography with a solution of iron (III) chloride to determine the consumption of the starting aldehyde. Then, the temperature was increased to 140 °C for decarboxylation. This step was also monitored using thin-layer chromatography. After completion, a small amount of acetone was added to solubilize the reaction mixture, which was then transferred into a saturated solution of sodium bicarbonate. The product was extracted using ethyl acetate (5 × 20 mL). The organic layer was washed with brine, dried with Na_2_SO_4_, filtered, and the solvent evaporated was in vacuo [18]. ¹H NMR (400 MHz, CDCl_3_) δ 3.86 (s, 3H), 4.01 (s, 3H), 6.21 (d, *J* 9.0 Hz, 1H), 6.59 (s, 1H), 7.54 (d, *J* 9.0 Hz, 1H); ^13^C NMR (100 MHz, DMSO-*d*_6_) δ 56.2, 60.8, 105.9, 109.5, 112.8, 134.2, 144.5, 145.9, 146.9, 149.6, 164.2. Yield: 60%.

#### 2.2.4. €-7-((3,7-Dimethylocta-2,6-dien-1-yl)oxy)-6,8-dimethoxy-2H-chromen-2-one (ACS51)

K_2_CO_3_ (1.2 eq) and geranyl bromide (1.0 eq) were added to a solution of isofraxidin (1.0 eq) in acetone (5 mL), and the mixture was stirred under reflux until completion. The product was extracted using ethyl acetate (5 × 20 mL). The organic layer was washed with brine, dried with Na_2_SO_4_, filtered, and the solvent was evaporated in vacuo. ¹H NMR (400 MHz, CDCl_3_) δ 1.59 (s, 3H), 1.66 (s, 3H), 1.69 (s, 3H), 2.05 (m, 4H), 3.89 (s, 3H), 4.03 (s, 3H), 4.68 (d, *J* 7 Hz, 2H), 5.06 (t, *J* 8 Hz, 1H), 5.55 (t, *J* 8 Hz, 1H), 6.35 (d, *J* 8 Hz, 1H), 6.66 (s, 1H), 7.60 (d, *J* 12 Hz, 1H); ^13^C NMR (100 MHz, CDCl_3_) δ 16.4, 17.7, 25.7, 26.4, 39.6, 56.3, 61.7, 70.3, 103.5, 114.4, 115.1, 119.6, 123.8, 131.7, 141.8, 142.5, 143.0, 143.5, 144.9, 150.7, 160.6. Yield: 70%.

#### 2.2.5. 6,8-Dimethoxy-7-((3-methylbut-2-en-1-yl)oxy)-2H-chromen-2-one (ACS50)

K_2_CO_3_ (1.2 eq) and prenyl bromide (1.0 eq) were added to a solution of isofraxidin (1.0 eq) in acetone (5 mL) and the mixture was stirred under reflux until completion. The product was extracted using ethyl acetate (5 × 20 mL). The organic layer was washed with brine, dried with Na_2_SO_4_, filtered, and the solvent evaporated in vacuo. ¹H NMR (400 MHz, CDCl_3_) δ 1.71 (s, 3H), 1.77 (s, 3H), 3,89 (s, 3H), 4.03 (s, 3H), 4.65 (d, *J* 8 Hz, 2H), 5.56 (t, *J* 8 Hz, 1H), 6.35 (d, *J* 8 Hz, 1H), 6.67 (s, 1H), 7.63 (d, *J* 8 Hz, 1H); ^13^C NMR (100 MHz, CDCl_3_) δ 18.2, 26.0, 56.5, 62.0, 70.5, 103.8, 114.6, 115.3, 120.1, 139.5, 142.1, 143.2, 143.7, 150.9, 160.8. Yield: 77%.

#### 2.2.6. (E)-7-((3,7-Dimethylocta-2,6-dien-1-yl)oxy)-2H-chromen-2-one (ACS48)

K_2_CO_3_ (1.2 eq) and geranyl bromide (1.0 eq) were added to a solution of umbelliferone (1.0 eq) in acetone (5 mL), and the mixture was stirred under reflux until completion. The product was extracted using ethyl acetate (5 × 20 mL). The organic layer was washed with brine, dried with Na_2_SO_4_, filtered, and the solvent was evaporated in vacuo. ¹H NMR (400 MHz, CDCl_3_) δ 1.62 (s, 3H), 1.68 (s, 3H), 1.77 (s, 3H), 2.11 (m, 4H), 4.61 (d, J 6 Hz, 2H), 5.01 (m, 1H), 5.48 (m, 1H), 6.26 (d, *J* 8 Hz, 1H), 6.85 (m, 2H), 7.36 (d, *J* 8 Hz, 1H), 7.65 (d, *J* 9 Hz, 1H). ^13^C NMR (100 MHz, CDCl_3_) δ 17.0, 17.9, 25.9, 26.5, 39.7, 65.7, 101.8, 112.6, 113.2, 113.5, 118.6, 123.8, 128.9, 132.2, 142.6, 143.7, 156.1, 161.5, 162.4. Yield: 82%.

#### 2.2.7. 7-((3-Methylbut-2-en-1-yl)oxy)-2H-chromen-2-one (ACS47)

K_2_CO_3_ (1.2 eq) and prenyl bromide (1.0 eq) were added to a solution of umbelliferone (1.0 eq) in acetone (5 mL), and the mixture was stirred under reflux until completion. The product was extracted using ethyl acetate (5 × 20 mL). The organic layer was washed with brine, dried with Na_2_SO_4_, filtered, and the solvent was evaporated in vacuo. ¹H NMR (400 MHz, CDCl_3_) δ 1.77 (s, 3H), 1.82 (s, 3H), 4.59 (d, *J* 7 Hz, 2H), 5.48 (t, *J* 6 Hz, 1H), 6.25 (d, *J* 9 Hz, 1H), 6.86 (m, 2H), 7.36 (d, *J* 8 Hz, 1H). ^13^C NMR (100 MHz, CDCl_3_) δ 18.3, 25.8, 65.4, 101.6, 112.4, 113.0, 113.2, 118.6, 128.7, 139.3, 143.5, 155.9, 161.3, 162.1. Yield: 79%.

#### 2.2.8. (E)-7-((3,7-Dimethylocta-2,6-dien-1-yl)oxy)-4-methyl-2H-chromen-2-one (ACS52)

K_2_CO_3_ (1.2 eq) and geranyl bromide (1.0 eq) were added to a solution of 4-methylumbelliferone (1.0 eq) in acetone (5 mL), and the mixture was stirred under reflux until completion. The product was extracted using ethyl acetate (5 × 20 mL). The organic layer was washed with brine, dried with Na_2_SO_4_, filtered, and the solvent was evaporated in vacuo. ¹H NMR (400 MHz, CDCl_3_) δ 1.62 (s, 3H), 1.68 (s, 3H), 1.77 (s, 3H), 2.11 (m, 4H), 2.41 (s, 3H), 4.61 (d, *J* 6 Hz, 2H), 5.01 (m, 1H), 5.48 (m, 1H), 6.14 (s, 1H), 6.83 (s, 1H), 6.87 (d, *J* 9 Hz,1H), 7.50 (d, *J* 9 Hz, 1H). ^13^C NMR (100 MHz, CDCl_3_) δ 16.8, 17.7, 18.7, 25.7, 26.3, 39.5, 65.5, 101.6, 111.9, 112.9, 113.5, 118.5, 123.6, 125.4, 132.0, 142.3, 152.6, 155.3, 161.4, 162.0. Yield: 91%.

#### 2.2.9. 4-Methyl-7-((3-methylbut-2-en-1-yl)oxy)-2H-chromen-2-one (ACS54)

K_2_CO_3_ (1.2 eq) and prenyl bromide (1.0 eq) were added to a solution of 4-methylumbelliferone (1.0 eq) in acetone (5 mL), and the mixture was stirred under reflux until completion. The product was extracted using ethyl acetate (5 × 20 mL). The organic layer was washed with brine, dried with Na_2_SO_4_, filtered, and the solvent was evaporated in vacuo. ¹H NMR (400 MHz, CDCl_3_) δ 1.78 (s, 3H), 1.82 (s, 3H), 2.40 (s, 3H), 4.59 (d, *J* 7 Hz, 2H), 5.48 (t, *J* 6 Hz, 1H), 6.13 (s, 1H), 6.83 (s, 1H), 6.86 (d, *J* 7 Hz, 1H), 7.48 (d, *J* 9 Hz, 1H). ^13^C NMR (100 MHz, CDCl_3_) δ 18.3, 18.7, 25.8, 65.4, 101.6, 111.8, 112.9, 113.5, 118.7, 125.5, 139.2, 152.6, 155.2, 161.4, 161.9. Yield: 86%.

#### 2.2.10. (E)-7-((3,7-Dimethylocta-2,6-dien-1-yl)oxy)-3,4,8-trimethyl-2H-chromen-2-one (ACS55)

K_2_CO_3_ (1.2 eq) and prenyl bromide (1.0 eq) were added to a solution of 7-hydroxy-3,4,8-trimethylcoumarin (1.0 eq) in acetone (5 mL), and the mixture was stirred under reflux until completion. The product was extracted using ethyl acetate (5 × 20 mL). The organic layer was washed with brine, dried with Na_2_SO_4_, filtered, and the solvent was evaporated in vacuo. ¹H NMR (400 MHz, CDCl_3_) δ 1.59 (s, 3H), 1.65 (s, 3H), 1.73 (s, 3H), 2.08 (m, 4H), 2.16 (s, 3H), 2.29 (s, 3H), 2.34 (s, 3H), 4.60 (d, *J* 6 Hz, 2H), 5.07 (m, 1H), 5.46 (m, 1H), 6.79 (d, *J* 9 Hz,1H), 7.36 (d, *J* 9 Hz, 1H). ^13^C NMR (100 MHz, CDCl_3_) δ 8.5, 13.4, 15.3, 17.0, 17.9, 25.9, 26.5, 39.7, 65.9, 108.2, 114.3, 114.5, 118.8, 119.7, 122.1, 123.9, 132.1, 141.4, 146.6, 151.4, 158.8, 162.9. Yield: 85%.

#### 2.2.11. 3,4,8-Trimethyl-7-((3-methylbut-2-en-1-yl)oxy)-2H-chromen-2-one (ACS56)

K_2_CO_3_ (1.2 eq) and geranyl bromide (1.0 eq) were added to a solution of 7-hydroxy-3,4,8-trimethylcoumarin (1.0 eq) in acetone (5 mL), and the mixture was stirred under reflux until completion. The product was extracted using ethyl acetate (5 × 20 mL). The organic layer was washed with brine, dried with Na_2_SO_4_, filtered, and the solvent was evaporated in vacuo. ¹H NMR (400 MHz, CDCl_3_) δ 1.71 (s, 3H), 1.77 (s, 3H), 2.18 (s, 3H), 2,31 (s, 3H), 2.36 (s, 3H), 4.60 (d, *J* 7 Hz, 2H), 5.50 (t, *J* 6 Hz, 1H), 6.83 (d, *J* 9 Hz, 1H), 7.40 (d, *J* 9 Hz, 1H). ^13^C NMR (100 MHz, CDCl_3_) δ 8.3, 13.2, 15.1, 18.3, 25.8, 65.6, 108.0, 114.1, 114.3, 118.6, 119.6, 121.9, 138.0, 146.4, 151.2, 158.6, 162.7. Yield: 83%.

### 2.3. Biological Assays

#### 2.3.1. Strains

In this study, four mutant strains of *S*. *cerevisiae* were used. These strains were kindly provided by Dr. Richard Cannon and Dr. Brian Monk (University of Otago, Dunedin, New Zealand). Firstly, a null mutant was constructed by deleting genes related to ABC transporters (Pdr5p, Yor1p, Snq2p, Pdr10p, Pdr11p, and Ycf1p). Then, three fluconazole-resistant strains were created by heterologous expression of transporters originally found in *Candida* spp., as summarized in Table 1 [19]. Also, two fluconazole-resistant *C. albicans* clinical isolates were used. While 95-142 overexpresses the ABC transporters, CaCdr1p and CaCdr2p [5], the PRI strain overexpresses the MFS transporter CaMdr1p [20]. The 95-142 strain was kindly provided by Dr. Theodore White (University of Missouri, Kansas City, MO, USA). Additionally, one fluconazole-sensitive *C. albicans* clinical isolate was used (ATCC 10231; American Type Culture Collection^TM^, Manassas, VA, USA). The strains were grown in YPD medium (2% glucose, 1% yeast extract, 2% peptone) at 30 °C (*S. cerevisiae*) or 37 °C (*C. albicans)* via agitation and were harvested in the exponential phase of growth whenever experiments were about to be performed.

#### 2.3.2. Antifungal Activity of Coumarins against *C. albicans* and *S. cerevisiae* Strains

Briefly, 5 × 10^3^ cells/mL of *C. albicans* strains (95-142, PRI and ATCC 10231) and 2 × 10^4^ cells/mL of the *S. cerevisiae* null mutant were incubated in RPMI-1640 (Sigma-Aldrich Co., St. Louis, MO, USA) at 37 °C or YPD medium at 30 °C, respectively, for 48 h via agitation (75 rpm) in the presence of 100 μg/mL of the compounds [21]. Cell growth was measured at 600 nm (Fluostar Optima, BMG Labtech, Offenburg, Germany).

#### 2.3.3. Antifungal Activity of Coumarins Combined with Fluconazole against *S. cerevisiae* Strains

Briefly, 2 × 10^4^ cells/mL of *S. cerevisiae* mutant strains (CaCdr1p+, CaCdr2p+, and CaMdr1p+) were incubated in YPD medium at 30 °C for 48 h using agitation (75 rpm), in the presence of 100 μg/mL of the coumarins with or without fluconazole at sub-inhibitory concentrations (125 μg/mL, 15.6 μg/mL and 15.6 μg/mL, respectively) [21]. Cell growth was measured at 600 nm (Fluostar Optima, BMG Labtech, Offenburg, Germany).

#### 2.3.4. Synergism Evaluation through Checkerboard Assay

This assay was conducted with the compounds active against *S. cerevisiae* in the presence of fluconazole. Briefly, 2 × 10^4^ cells/mL of *S. cerevisiae* mutant strains (CaCdr1p+, CaCdr2p+, and CaMdr1p+) were incubated in YPD medium at 30 °C for 48 h with agitation (75 rpm), in the presence of serial concentrations of the coumarins (100–6.25 μg/mL) and fluconazole (250–3.91 μg/mL). Cell growth was measured at 600 nm (Fluostar Optima, BMG Labtech, Offenburg, Germany). Serial dilutions of the coumarins and fluconazole alone were made to obtain the minimum inhibitory concentration (MIC) of each compound. The interaction between the coumarins and fluconazole was assessed using the fractional inhibitory concentration index (FICI). The FICI is calculated by summing the fractional inhibitory concentration (FIC) of each drug, and the FIC is the ratio of the MIC of a drug combined with a second drug and the MIC of a drug alone. Synergistic, additive, indifferent, and antagonistic interactions correspond to FICI ≤ 0.5, >0.5–1.0, 1.0–4.0, and >4.0, respectively [21].

After evaluating the association between coumarins and fluconazole against *S. cerevisiae* strains, the combinations that presented either additive or synergistic interactions were tested against the growth of *C. albicans*. The compounds active against CaCdr1p+ or CaCdr2p+ were tested against the 95-142 strain, while the compounds active against CaMdr1p+ were tested against the PRI strain. Briefly, 5 × 10^3^ cells/mL of *C. albicans* resistant strains was incubated in RPMI-1640 medium at 37 °C for 48 h with agitation (75 rpm) in the presence of serial concentrations of the coumarins (100–6.25 μg/mL) and fluconazole (1000–15.6 μg/mL). Cell growth was measured at 600 nm (Fluostar Optima, BMG Labtech, Offenburg, Germany), and the FICI values were calculated using the aforementioned method.

#### 2.3.5. Erythrocyte Viability

The effect of the compounds on erythrocyte viability was assessed as described elsewhere [22]. Cells were washed three times and resuspended in phosphate-buffered saline (PBS) to a final of concentration of 2% v/v. Then, cells were incubated in the presence of the compounds at 100 μg/mL for 60 min at 37 °C. Afterward, cells were harvested by centrifugation at 3000× *g* for 5 min and 1 mL of supernatant was transferred to glass cuvettes. The absorbance of hemoglobin released from erythrocytes was measured at 540 nm (SP220, Biospectro, Curitiba, Brazil). Controls of 100% and 0% hemolysis were performed by incubating the cells in PBS in the presence or absence of 1% Triton X-100, respectively. Also, controls with DMSO and acetonitrile were performed.

#### 2.3.6. In Silico Toxicity Prediction

The toxicity of the coumarins was predicted in silico using OSIRIS Property Explorer [23], which provides information regarding mutagenic, tumorigenic, irritant, and reproductive effects, and GUSAR (General Unrestricted Structure–Activity Relationships), which provides a LD_50_ (lethal dose to 50% of a given population) of the substances in rats, considering four routes of administration (intraperitoneal, intravenous, oral, and subcutaneous) [24].

#### 2.3.7. Statistical Analysis

All the experiments were performed at least three times, and the results were expressed as mean ± standard deviation. Data were analyzed using Student’s *t* test, and *p* values lower than 0.05 were considered significant.

## 3. Results

### 3.1. Synthesis

**Figure 2:**
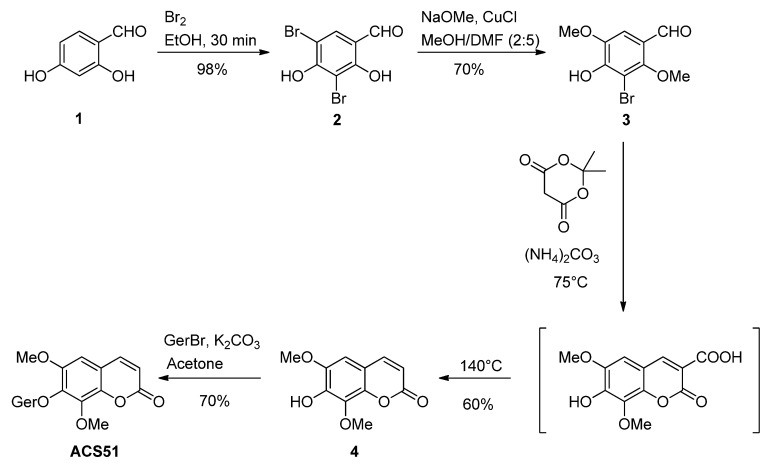
Total synthesis of altissimacoumarin D in four steps.

The synthesis of altissimacoumarin D is summarized in Figure 2. This coumarin was first isolated by Dao et al. in 2012 [16], and the first total synthesis performed by our group [17] involved the alkylation of isofraxidin, another natural occurring coumarin. In this study, our goal was to improve that first synthesis via the most crucial step, the formation of the coumarin ring. The first two steps of the synthesis, which lead to the formation of the methoxylated intermediate, were kept the same, since the yields were already improved, and the purification process was simple.

Attempts were made to form the coumarin ring in a way that would be more efficient, greener and easier to purify. Then, the reaction was performed as described by Schijndel et al. [18], with slight modifications. Instead of forming the coumarin ring using malonic acid, 2-hydroxybenzadehyde and ammonium bicarbonate, we used ammonium carbonate, 2,4-dihydroxy-3,5-dimethoxybenzaldehyde and Meldrum’s acid with no solvent in this study. Isofraxidin was obtained with a yield of 60%.

After obtaining isofraxidin, the next step was to obtain altissimacoumarin D via an alkylation. In our previous study, this step was performed via a Mitsunobu reaction that, although efficient, required the use of a dry solvent, a 24 h reaction, and made the purification of the product challenging. Thus, in this study, we performed the alkylation via a simpler methodology using acetone, potassium carbonate and geranyl bromide; altissimacoumarin D was obtained with a yield of 70%.

**Figure 3:**
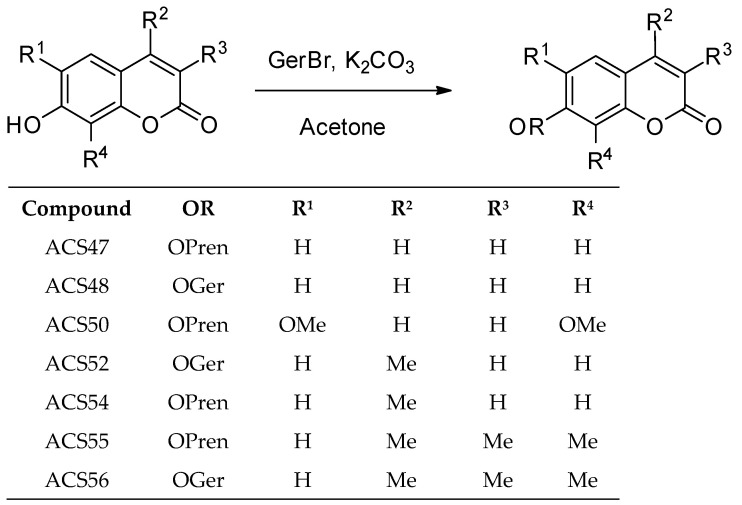
Analogues of altissimacoumarin D obtained through the alkylation of commercially available compounds. OMe—methoxy group; Me—methyl group; OGer—geranyl group; OPren—prenyl group.

Since only one biological test was performed with altissimacoumarin D [16], and the compound was shown to be promising due to its prenylated coumarin nature, seven other coumarins with different substitution patterns were synthesized, varying the alkyl chain between geranyl and prenyl. Figure 3 shows the structures of the analogues of altissimacoumarin D that were synthesized in this study.

### 3.2. Antifungal Activity of Coumarins against C. albicans and S. cerevisiae Strains

**Figure 4:**
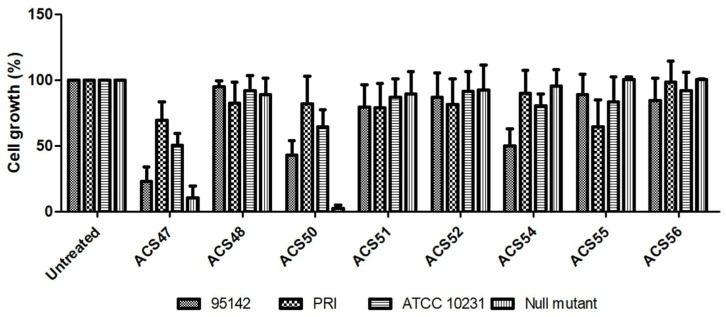
Antifungal activity screening against *C. albicans* and *S. cerevisiae* strains. Cells (95-142, PRI, ATCC 10231 and Null mutant) were incubated with 100 μg/mL of coumarins for 48 h. Cell growth was measured at 600 nm. Data are expressed as mean ± standard deviation of three independent experiments.

Altissimacoumarin D and the coumarin analogues were screened for antifungal activity against *C. albicans* and *S. cerevisiae* strains (Figure 4).

While the natural product was inactive at the tested concentration, ACS47, ACS50, and ACS54 at 100 μg/mL presented antifungal activity. ACS47 inhibited the growth of 95-142, PRI, ATCC 10231, and Null mutant strains by 77.2%, 30.4%, 49.5%, and 89.8%, respectively. ACS50 inhibited the growth of 95-142, ATCC 10231 and Null mutant strains by 57%, 35.5%, and 97.6%, respectively. ACS54 inhibited the growth of the 95-142 strain by 50.2%.

### 3.3. Antifungal Activity of Coumarins Combined with Fluconazole against S. cerevisiae Strains

**Figure 5:**
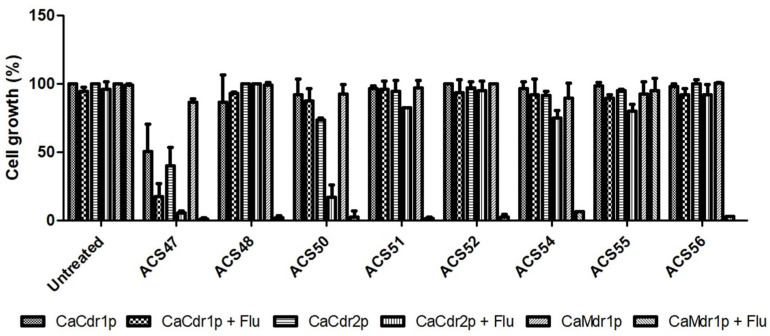
Antifungal activity of coumarins combined with fluconazole against *S. cerevisiae* strains. Cells (CaCdr1p+, CaCdr2p+, and CaMdr1p+) were incubated with 100 μg/mL of coumarins and sub-inhibitory concentrations of fluconazole for 48 h. Cell growth was measured at 600 nm. Data are expressed as the mean ± standard deviation of three independent experiments.

To assess if the compounds inhibit *C. albicans* efflux pumps, their antifungal activity alone and combined with fluconazole were evaluated against *S. cerevisiae* mutant strains that overexpress these transporters. Compounds ACS48, ACS51, ACS52, ACS54, ACS55 and ACS56 did not exhibit antifungal activity alone. On the other hand, ACS47 inhibited the growth of CaCdr1p+ and CaCdr2p+ by 49.6% and 50.3%, respectively, and ACS50 inhibited the growth of CaCdr2p+ by 26.5%. However, in combination with fluconazole, all compounds, except for ACS55, inhibited the growth of CaMdr1p+ by 93.6–99.1%. Moreover, the combination of ACS47 with fluconazole inhibited the growth of CaCdr1p+ and CaCdr2p+ by 82.5% and 94.7%, respectively, while the combination of ACS50 and fluconazole inhibited the growth of CaCdr2p+ by 83.2% (Figure 5).

### 3.4. Synergism Evaluation through Checkerboard Assay

Since the coumarins presented antifungal activity when combined with fluconazole, a checkerboard assay was performed to determine the type of interaction between the compounds. Firstly, the *S. cerevisiae* mutant strains were used. As shown in Table 2, ACS47 at 50 μg/mL was synergistic with fluconazole against CaCdr1p+ and CaCdr2p+, reducing the fluconazole MIC by 4–8 fold. The interaction between ACS50 (at 25 μg/mL) and fluconazole was classified as additive, and the coumarin halved fluconazole MIC against CaCdr2p+. All the coumarins were able to diminish fluconazole MIC against CaMdr1p+. Compounds ACS47–54 displayed synergistic activity with fluconazole, with FICI values ranging from 0.188 to 0.500. ACS55 and ACS56 presented an additive interaction with fluconazole.

Due to these positive results, the checkerboard assay was also performed using *C. albicans* strains. All the compounds were tested against PRI because this strain overexpresses CaMdr1p. Furthermore, ACS47 and ACS50 were tested against 95-142, a *C. albicans* strain that overexpresses both CaCdr1p and CaCdr2p. At 50 μg/mL, ACS 47 and ACS50 reduced fluconazole MIC against 95-142 by 4–8 fold, with FICI values of 0.375 and 0.500, respectively. Against the PRI strain, only ACS47 and ACS50 showed a combined activity with fluconazole. Both compounds reduced the fluconazole MIC from 1000 μg/mL to 15.6 μg/mL (Table 3).

### 3.5. Erythrocyte Viability

**Figure 6:**
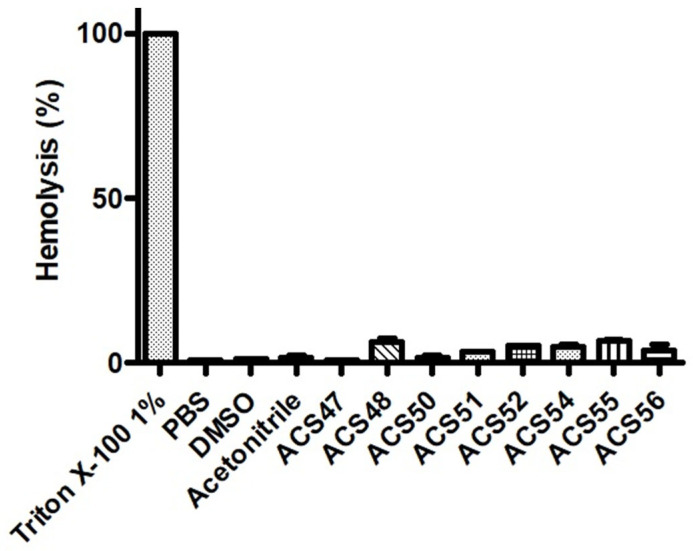
Hemolysis assay. Erythrocytes were incubated in the presence of coumarins, and the absorbance of released hemoglobin was measured at 540 nm. Triton X-100 and PBS were used as controls of 100% and 0% hemolysis, respectively. DMSO and acetonitrile were also used as controls. Data are expressed as mean ± standard deviation of three independent experiments. PBS: phosphate-buffered saline. DMSO: dimethyl sulfoxide.

At 100 μg/mL, the compounds exerted 0.8–6.6% of hemolysis. The lowest hemolytic compounds were ACS47 and ACS50, which presented results comparable to PBS and DMSO controls (Figure 6).

### 3.6. In Silico Toxicity Prediction

The toxicity of the prenylated coumarins was predicted in silico using two free software programs. According to OSIRIS Property Explorer, ACS47 and ACS50 were predicted not to be mutagenic, tumorigenic and irritant; they do not have any reproductive effects on humans, a toxicity profile similar to fluconazole. ACS48 and ACS51 may be irritants, while ACS54 may induce reproductive effects. Also, ACS52, ACS55, and ACS56 may be irritant and exert reproductive effects. None of the compounds are predicted to be mutagenic or tumorigenic (Table 4).

Data obtained by the GUSAR software is summarized in Table 5. Coumarins intraperitoneal, intravenous, oral, and subcutaneous LD_50_ ranged from 333.1 to 581 mg/kg, 53.41 to 121.6 mg/kg, 1057 to 2690 mg/kg, and 845.7 to 2462 mg/kg, respectively. Fluconazole LD_50_ ranged from 200.4 mg/kg to 708 mg/kg.

## 4. Discussion

### 4.1. Synthesis of Prenylated Coumarins

Natural products have been extensively explored as potential sources of new drugs, due to their wide variety of pharmacological activities. The Chinese tree *A. altissima*, for example, possesses compounds with anti-inflammatory [25], anticancer [26], and neuroprotective [27] properties. Also, Li et al. (2021) isolated an alkaloid with antifungal activity against *Fusarium oxysporum* [28], and Dao et al. (2012) purified terpenylated coumarins with potential anticancer activity from *A. altissima* [16]. Coumarins are considered privileged scaffolds for the synthesis of potential drugs with higher yields when compared to isolation from natural sources [29]. The presence of terpenylated coumarins in *A. altissima*, the feasibility of their synthesis, their potential pharmacological activity, and the need to discover substances that reverse antifungal resistance in *Candida* spp. encouraged the synthesis and evaluation of antifungal activity of altissimacoumarin D, the main compound of the class found in *A. altissima* and its analogues. In the present study, isofraxidin was efficiently prepared in a one-pot solvent-free reaction. Also, the Mitsunobu reaction, which was a different approach from the previous synthesis of altissimacoumarin D that we reported, was not used in this study [17]. These modifications led to a lower use of organic solvent to obtain the desired coumarins, without worsening the yields of the reactions.

### 4.2. Antifungal Activity of Coumarins against C. albicans and S. cerevisiae Strains

After preparing the coumarins, their ability to inhibit the growth of fluconazole-sensitive and fluconazole-resistant strains was evaluated. Only three compounds (ACS47, ACS50, and ACS54) presented antifungal activity, and all of them are substituted with a prenyl group. Their geranyl-substituted counterparts (ACS48, ACS51, and ACS52, respectively) did not display antifungal activity. ACS47 was active against the four strains tested, while ACS50 and ACS54 inhibited the growth of three strains and one strain, respectively. It may be observed that the insertion of methyl groups into the benzopyran moiety decreased the antifungal activity of the compounds, which may explain why ACS55 was not active against the yeasts, since it bears three methyl groups. ACS47 and ACS50 differ due to the presence of two methoxy substitutions in the latter. Results show that this substitution pattern does not influence the antifungal activity of the compound. Kurdelas et al. (2010) evaluated the antifungal activity of three prenylated coumarins with geranyl groups (auraptene, 5′-hydroxy auraptene, and 5′-oxo-auraptene) against yeasts and filamentous fungi and observed that none of the substances inhibited the growth of *C. albicans*, *C. tropicalis*, and *S. cerevisiae* [30], such as in this study. Jia et al. (2019) observed that coumarin (1,2-benzopyrone) exerts anti-*C. albicans* activity at 0.5 mg/mL–2 mg/mL by inducing apoptosis [31]. In this study, coumarins inhibited *C. albicans* and *S. cerevisiae* growth at 0.1 mg/mL. This action may be related to apoptosis induction, as seen in the unsubstituted coumarin, and the prenyl substitution could ease the interaction between the coumarins and plasma membrane, explaining the antifungal activity observed at a lower concentration [32].

### 4.3. Antifungal Activity of Coumarins Combined with Fluconazole against S. cerevisiae Strains

To evaluate whether the compounds inhibit *C. albicans* efflux pumps, hence precluding multidrug resistance and sensitizing the fungi to fluconazole, a screening assay using *S. cerevisiae* mutant strains was performed. Each strain heterologously expresses a specific MDR transporter, making it possible to comprehend exactly which efflux pumps the coumarins may affect. All coumarins, except for ACS55, improved the fluconazole antifungal activity against the *S. cerevisiae* strain that overexpresses CaMdr1p, an MFS transporter. This finding indicates that the compounds are able to inhibit this specific efflux pump, regardless of whether the coumarin possesses a prenyl or a geranyl substitution. ACS55, the only coumarin that did not affect CaMdr1p in this assay, bears three methyl groups that may impair its binding to the transporter. Moreover, data show that ACS47 and ACS50 also inhibit ABC transporters. These two coumarins carry a prenyl-substitution and are unsubstituted in the lactone ring. The presence of one methyl group is the only difference between ACS47, which inhibits CaCdr1p and CaCdr2p, as well as ACS54, which did not affect ABC transporters. In a previous study, it was observed that beta-lapachone impaired CgCdr2p ATPase activity, while beta-nor-lapachone did not have an inhibitory activity, and the difference between these two naphthoquinones is a methylene group [21]. Esposito et al. (2017) also observed that minor modifications in the structures of jatrophanes modified their inhibitory activity against CaCdr1p and CaMdr1p [33]. These data suggest that small increases in substitution may impact the inhibition of *Candida* spp. efflux transporters, which is important to optimize the design of new MDR inhibitors. The results of the screening assay were confirmed using a checkerboard. Eight out of the eleven tested combinations displayed synergism, and compounds ACS47 and ACS54 were the most active substances. Also, when comparing FICI values, it was not possible to determine a link between the presence of prenyl or geranyl substitutions and the inhibitory activity of the coumarins on the MFS transporter. To our knowledge, there are no reports on the ability of prenylated coumarins to inhibit *Candida* efflux pumps. However, the effects of geraniol have already been described. Singh et al. (2018) assessed the influence of the monoterpene on CaCdr1p and CaMdr1p, and observed that it affects only the ABC transporter. In silico analysis revealed that geraniol binds to CaCdr1p via hydrophobic interactions and H-bonds, while the control compound farnesol binds to the efflux pump only via hydrophobic interactions. Also, geraniol presented a lower binding energy than farnesol, indicating a higher affinity to the active site of CaCdr1p [34]. ACS47 and ACS50 are prenyl-substituted, and it may be hypothesized that the reduced size of the prenyl side chain, in comparison to a geranyl or a farnesyl chain, would increase the affinity of the coumarins to CaCdr1p, explaining the ability of these specific two compounds to inhibit ABC transporters. Aside from geraniol, the inhibitory activity of coumarin scopoletin on efflux pumps was also evaluated [35]. This substance is an unprenylated coumarin, presenting one hydroxyl and one methoxy substitution. Scopoletin exerted an antifungal effect on *C. tropicalis*, and at a sub-inhibitory concentration, promoted a four-fold decrease in fluconazole MIC, an effect related to the inhibition of efflux pumps. Although geraniol and scopoletin separately modulate *Candida* spp. efflux pumps, the geranylated coumarins tested in this study were unable to inhibit ABC transporters. One of the substances tested, ACS48 (also known as auraptene), induced the expression of P-glycoprotein in human intestinal cells [36]. Since this efflux pump is analogous to CaCdr1p and CaCdr2p, ACS48 and the other coumarins could induce the expression of *CDR1 and CDR2* instead of inhibiting the transporters, which may explain the lack of activity in these substances combined with fluconazole against *S. cerevisiae* mutant strains that overexpress ABC transporters.

### 4.4. Antifungal Activity of Coumarins Combined with Fluconazole against Strains of C. albicans 

The *S. cerevisiae* mutant strains used in this study are important tools to assess the action of substances in individual efflux pumps. Nonetheless, to endorse a possible application of these compounds combined with fluconazole, it is essential to test them against pathogenic strains. Thus, fluconazole-resistant *C. albicans* clinical strains were also employed in this study. Data show that none of the compounds were synergistic with fluconazole against PRI strain. It is important to consider that FICI is a parameter that may not be strictly followed. In this study, FICI of 0.516 were obtained using ACS47 and ACS50 combined with fluconazole and, according to these results, the interactions are additive. However, the fact that no synergism was observed does not disqualify these substances as promising candidates to be used in clinics. Both coumarins reduced fluconazole MIC by 64 times, reinforcing their ability to inhibit efflux pumps. Although the other six coumarins can also inhibit CaMdr1p, indifferent interactions were detected against PRI strain. These compounds present geranyl groups and/or substitutions in the lactone ring, increasing their hydrophobicity. Since the plasma membranes from *S. cerevisiae* and *C. albicans* differ in composition, the diffusion of more hydrophobic substances across the membrane may be hindered in *C. albicans* [37]. Therefore, the impossibility of concentrating in the cytoplasm may justify the lack of activity of some compounds, even when they are able to inhibit efflux pumps. This could explain why ACS47 and ACS50, two prenyl-substituted coumarins, performed better than the other substances. ACS47 and ACS50 also improved the antifungal activity of fluconazole against 95-142. This *C. albicans* strain overexpresses two ABC transporters, CaCdr1p and CaCdr2p, and ACS47 is able to block both of them. Interestingly, ACS50 inhibits only CaCdr2p, but synergized with fluconazole against 95-142. This could be explained by a possible lower expression of *CDR1* in comparison to *CDR2*. In this scenario, the rate of fluconazole extrusion by CaCdr1p would not be sufficient, and the inhibition of CaCdr2p would promote fluconazole intracellular accumulation, allowing the antifungal agent to exert its activity. Moraes et al. (2020) observed that the naphthoquinone beta-lapachone exhibits the same inhibitory pattern of ACS50, i.e., the compound inhibits only CaCdr2p but displays synergism with fluconazole against the 95-142 strain [38].

### 4.5. Toxicity

Aside from being effective in treating candidiasis, alone and/or combined with fluconazole, the prenylated coumarins tested in this study must be safe for human use to be good candidates as antifungal agents. In this study, in vitro and in silico assays were conducted to verify the toxicity of the coumarins. The substances had a low hemolytic effect, which is essential to ensure the safety of a substance, since drugs reach the bloodstream after administration to the infection site. The lack of in vitro toxicity corroborates the findings of Maleki et al. (2019), who assessed the in vitro toxicity of O-prenylated coumarins against an HDF cell line and observed that ACS47, ACS48 and umbelliprenin did not affect its viability [39].

Although the absence of hemolysis is an important feature of any drug, it may be considered an initial test to assess toxicity [40]. Other cells constitute the human body, and a non-hemolytic drug may not be exempt from toxic effects. To better understand the toxicity profile of the coumarins, an in silico analysis was performed. None of the compounds showed mutagenic or tumorigenic effects. However, eight compounds were predicted to be irritant, and four compounds may have reproductive effects. Comparing the compounds, it is possible to observe that the presence of three methyl groups in the coumarin moiety induces irritant effects, regardless of the presence of a prenyl or a geranyl substitution. Among the coumarins with less substitutions, the prenylated coumarins do not have irritant effects, while their geranylated counterparts are toxic. On the other hand, all the compounds that bear a methyl group in the lactone ring cause reproductive effects, aside from the presence of a prenyl or geranyl substitution. The toxicity profiles predicted for ACS47 and ACS50 are similar to that observed for fluconazole, reinforcing a possible role of these two coumarins as new anti-*Candida* agents. The in silico analysis also provided LD_50_ for different routes of administration. The intraperitoneal and intravenous LD_50_ values for all coumarins were lower than those obtained for fluconazole. Contrarily, the coumarins presented higher oral and subcutaneous LD_50_ values than fluconazole. These results corroborate the lack of toxicity and encourage future studies with animals to test the in vivo activity of the coumarins against *C. albicans.*

## 5. Conclusions

In this study, we were able to improve the synthesis of altissimacoumarin D. Using a smaller amount of organic solvent, altissimacoumarin D and analogues were obtained in good yields and purity. ACS47, ACS50 and ACS54 presented antifungal activity alone, while all the coumarins (except for ACS55) inhibited at least one efflux pump. ACS48, ACS51, ACS52, ACS54 and ACS56 inhibited only CaMdr1p. No classic CaMdr1p inhibitors are available to be used as positive controls in experiments, and these coumarins could be used to fill this gap. ACS47 and ACS50 have synergistic activity when combined with fluconazole against the growth of *Candida albicans*. This effect is related to the inhibition of the efflux pumps CaCdr1p, CaCdr2p, and/or CaMdr1p. ACS47 inhibits all three transporters, which is important considering that the goal is to develop a new antifungal treatment since the substance is able to enhance fluconazole activity regardless of the efflux pump related to the resistance. Also, these compounds did not present toxicity in vitro and in silico. Therefore, ACS47 and ACS50 emerge as potential candidates for use as adjuvants in candidiasis therapy caused by resistant strains.

## Figures and Tables

**Table 1:** Strains used in this study.

Strain	Efflux Transporter	Family	Species
Null mutant	None	-	*S. cerevisiae*
CaCdr1p+	CaCdr1p	ABC	*S. cerevisiae*
CaCdr2p+	CaCdr2p	ABC	*S. cerevisiae*
CaMdr1p+	CaMdr1p	MFS	*S. cerevisiae*
95-142	CaCdr1p and CaCdr2p	ABC	*C. albicans*
PRI	CaMdr1p	MFS	*C. albicans*
ATCC 10231	None	-	*C. albicans*

**Table 2:** Checkerboard assays of coumarins against *S. cerevisiae* mutant strains.

Strain and Compound	Compound (µg/mL)			Fluconazole (µg/mL)				
	MIC^a^	MIC^b^	FIC	MIC^a^	MIC^b^	FIC	FICI	Outcome
CaCdr1p+								
ACS47	>100	50	0.250	>250	62.5	0.125	0.375	S
CaCdr2p+								
ACS47	>100	50	0.250	62.5	15.6	0.250	0.500	S
ACS50	>100	25	0.125	62.5	31.3	0.500	0.625	A
CaMdr1p+								
ACS47	>100	25	0.125	62.5	3.91	0.063	0.188	S
ACS48	>100	50	0.250	62.5	7.81	0.125	0.375	S
ACS50	>100	25	0.125	62.5	15.6	0.250	0.375	S
ACS51	>100	50	0.250	62.5	15.6	0.250	0.500	S
ACS52	>100	12.5	0.063	62.5	15.6	0.250	0.313	S
ACS54	>100	12.5	0.063	62.5	7.81	0.125	0.188	S
ACS55	>100	100	0.500	62.5	31.3	0.500	1.000	A
ACS56	>100	100	0.500	62.5	15.6	0.250	0.750	A

MIC^a^, MIC of compound alone; MIC^b^, MIC of combined compound; FIC, fractional inhibitory concentration; FICI, fractional inhibitory concentration index; S, synergism; A, additive.

**Table 3:** Checkerboard assays of coumarins against fluconazole-resistant clinical strains of *Candida albicans*.

Strain and Compound	Compound (µg/mL)			Fluconazole (µg/mL)				
	MIC^a^	MIC^b^	FIC	MIC^a^	MIC^b^	FIC	FICI	Outcome
95-142								
ACS47	>100	50	0.250	500	62.5	0.125	0.375	S
ACS50	>100	50	0.250	500	125	0.250	0.500	S
PRI								
ACS47	>100	100	0.500	1000	15.6	0.016	0.516	A
ACS48	>100	>100	1	1000	1000	1	2	I
ACS50	>100	100	0.500	1000	15.6	0.016	0.516	A
ACS51	>100	>100	1	1000	1000	1	2	I
ACS52	>100	>100	1	1000	1000	1	2	I
ACS54	>100	>100	1	1000	1000	1	2	I
ACS55	>100	>100	1	1000	1000	1	2	I
ACS56	>100	>100	1	1000	1000	1	2	I

MIC^a^, MIC of compound alone; MIC^b^, MIC of combined compound; FIC, fractional inhibitory concentration; FICI, fractional inhibitory concentration index; I, indifferent; A, additive.

**Table 4:** Toxicity risk prediction by Osiris Property Explorer.

Compound	Mutagenic	Tumorigenic	Irritant	Reproductive Effect
ACS47	No	No	No	No
ACS48	No	No	Yes	No
ACS50	No	No	No	No
ACS51	No	No	Yes	No
ACS52	No	No	Yes	Yes
ACS54	No	No	No	Yes
ACS55	No	No	Yes	Yes
ACS56	No	No	Yes	Yes

**Table 5:** Rat acute toxicity prediction by GUSAR (mg/kg).

Compound	Rat IP LD_50_	Rat IV LD_50_	Rat Oral LD_50_	Rat SC LD_50_
ACS47	476.4	65.99	1551	2462
ACS48	456.5	90.71	2157	1234
ACS50	426.8	93.38	1057	1461
ACS51	525	121.6	1401	845.7
ACS52	401.5	83.72	1979	918.6
ACS54	581.3	66.26	1470	2452
ACS55	333.1	53.41	2445	1116
ACS56	351.9	74.89	2690	948.3
Fluconazole	708	200.4	584.4	511.3

## Data Availability

Not applicable.

## Supplementary Materials

The following supporting information can be downloaded at: https://www.mdpi.com/article/10.3390/jof9070758/s1. Figure S1. ^1^H NMR spectra of ACS51; Figure S2. ^13^C NMR spectra of ACS51; Figure S3. ^1^H NMR spectra of ACS50; Figure S4. ^13^C NMR spectra of ACS50; Figure S5. ^1^H NMR spectra of ACS48; Figure S6. ^13^C NMR spectra of ACS48; Figure S7. ^1^H NMR spectra of ACS47; Figure S8. ^13^C NMR spectra of ACS47; Figure S9. ^1^H NMR spectra of ACS52; Figure S10. ^13^C NMR spectra of ACS52; Figure S11. ^1^H NMR spectra of ACS54; Figure S12. ^13^C NMR spectra of ACS54; Figure S13. ^1^H NMR spectra of ACS56; Figure S14. ^13^C NMR spectra of ACS56; Figure S15. ^1^H NMR spectra of ACS55; Figure S16. ^13^C NMR spectra of ACS55.
