# Supplementary material for: Synthesis of Altissimacoumarin D and Other Prenylated Coumarins and Their Ability to Reverse the Multidrug Resistance Phenotype in Candida albicans
Source: J Fungi (Basel). 2023 Jul 18;9(7):758. doi: 10.3390/jof9070758 (PMC10381857; doi:10.3390/jof9070758)

Synthesis of altissimacoumarin D and other prenylated coumarins and their ability to reverse the multidrug resistance phenotype in *Candida albicans*

Supplementary information

^1H and ^{13}C NMR spectra

Figure S1. ^{1}H NMR spectra of ACS51.

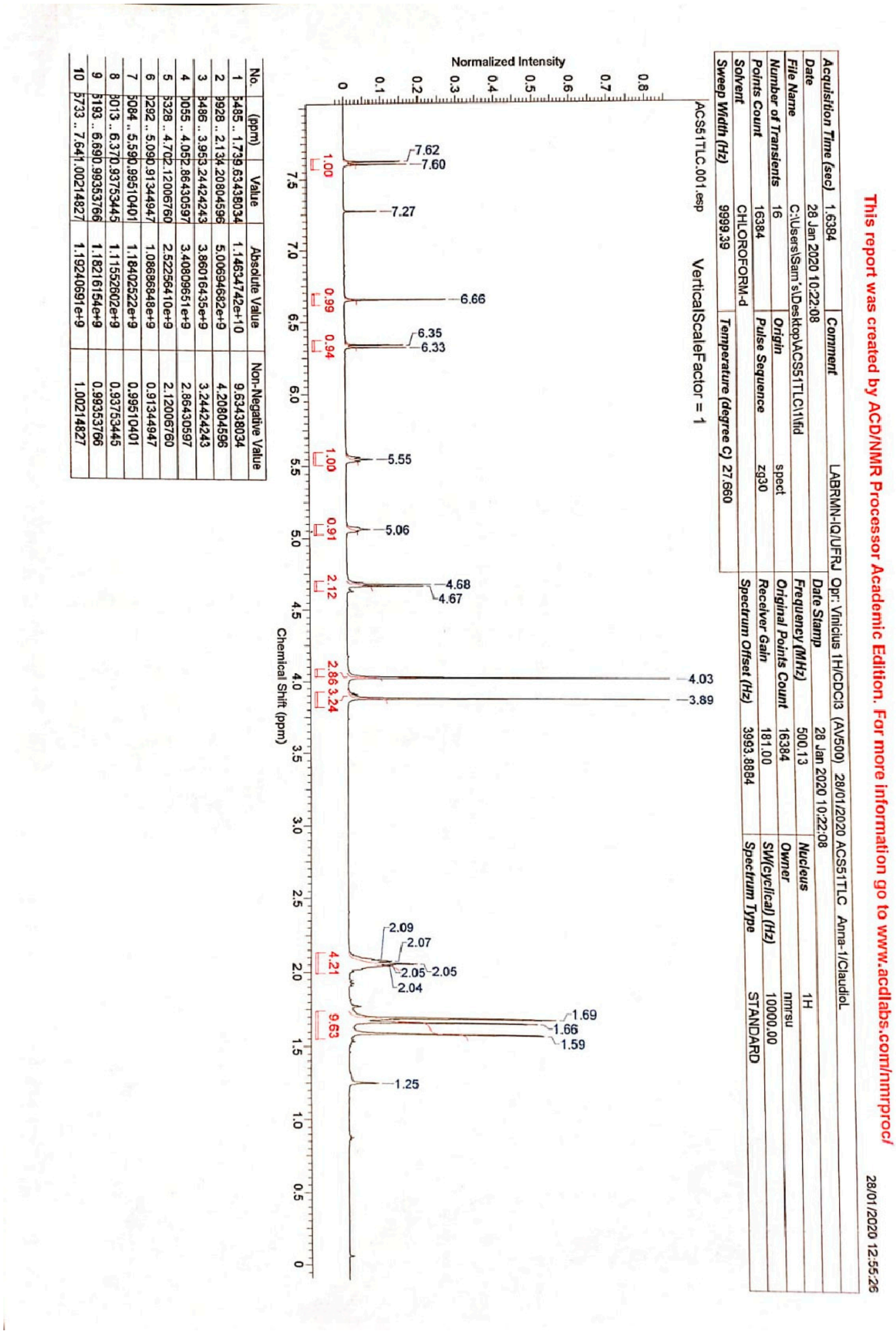

Figure S2. ^{13}C NMR spectra of ACS51.

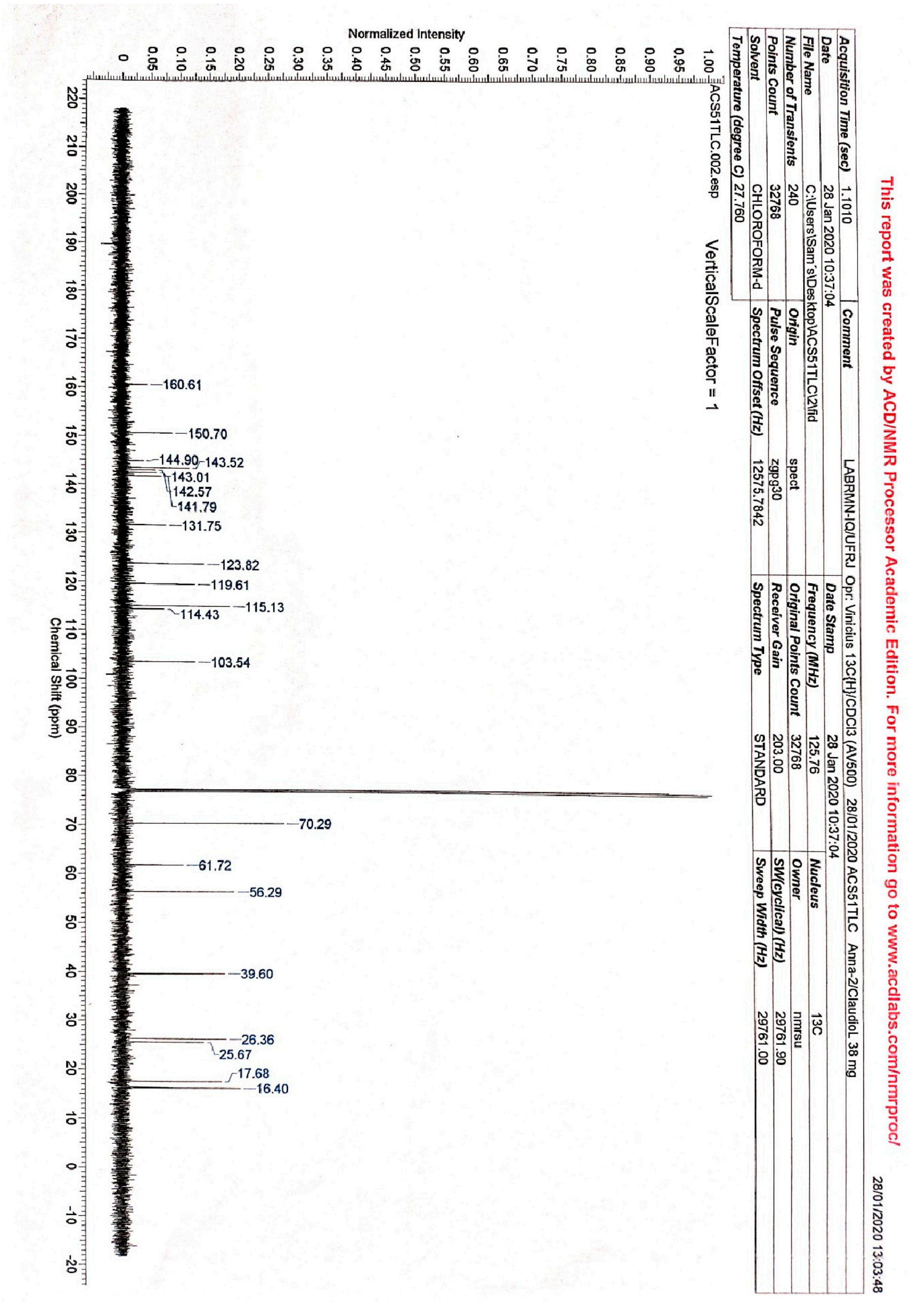

Figure S3. ^{1}H NMR spectra of ACS50.

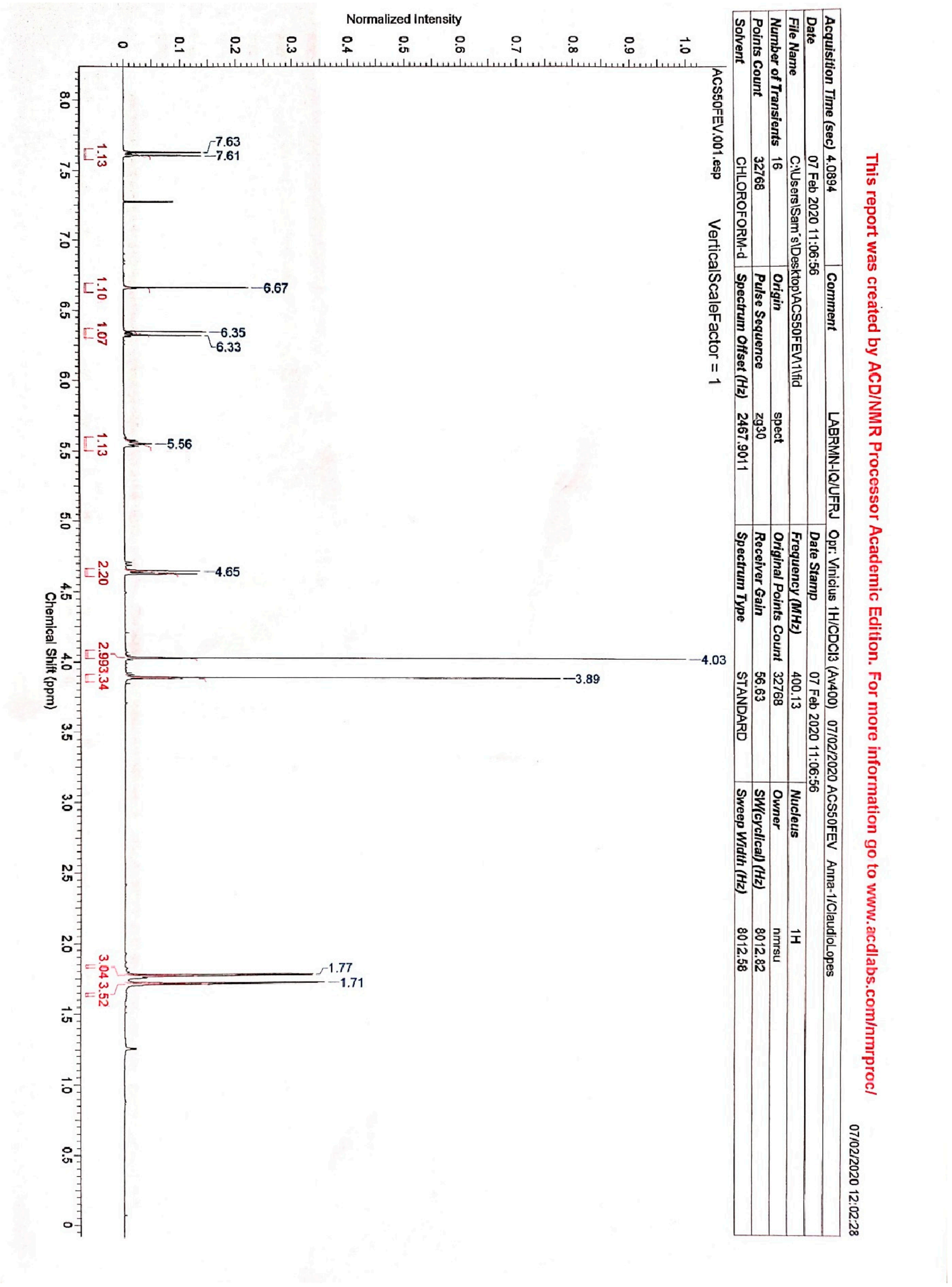

Figure S4. ^{13}C NMR spectra of ACS50.

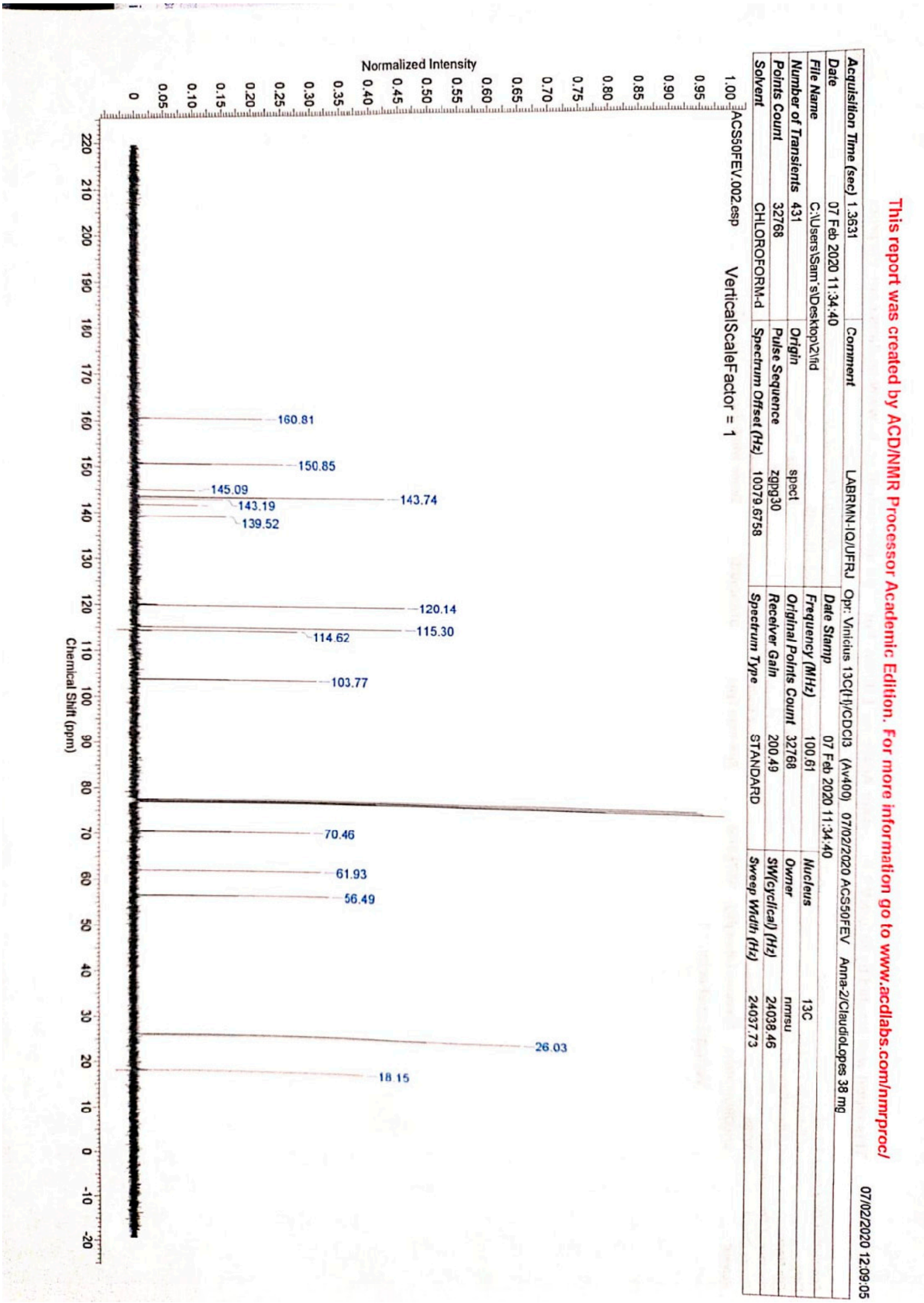

Figure S5. ^{1}H NMR spectra of ACS48.

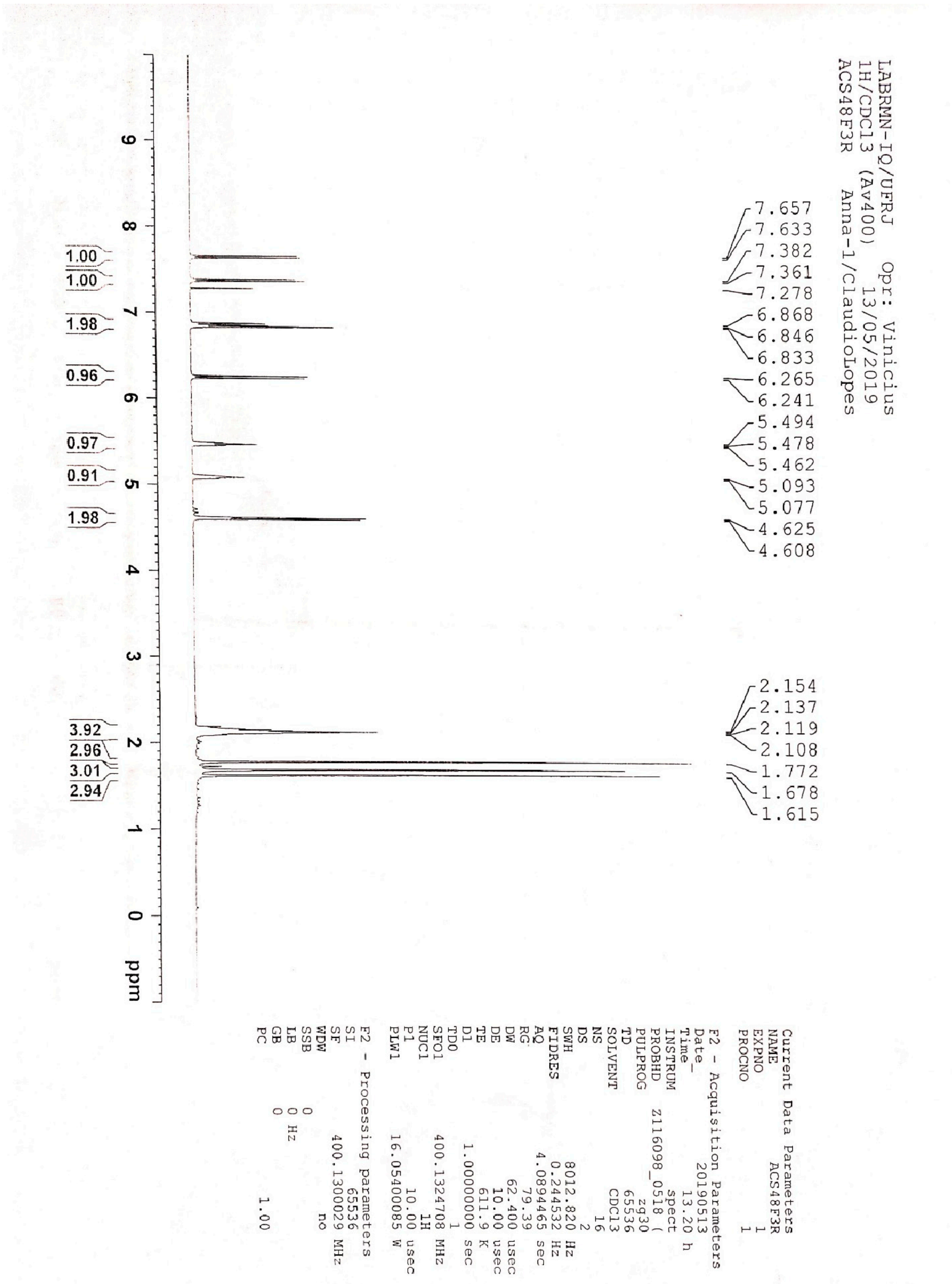

Figure S6. ^{13}C NMR spectra of ACS48.

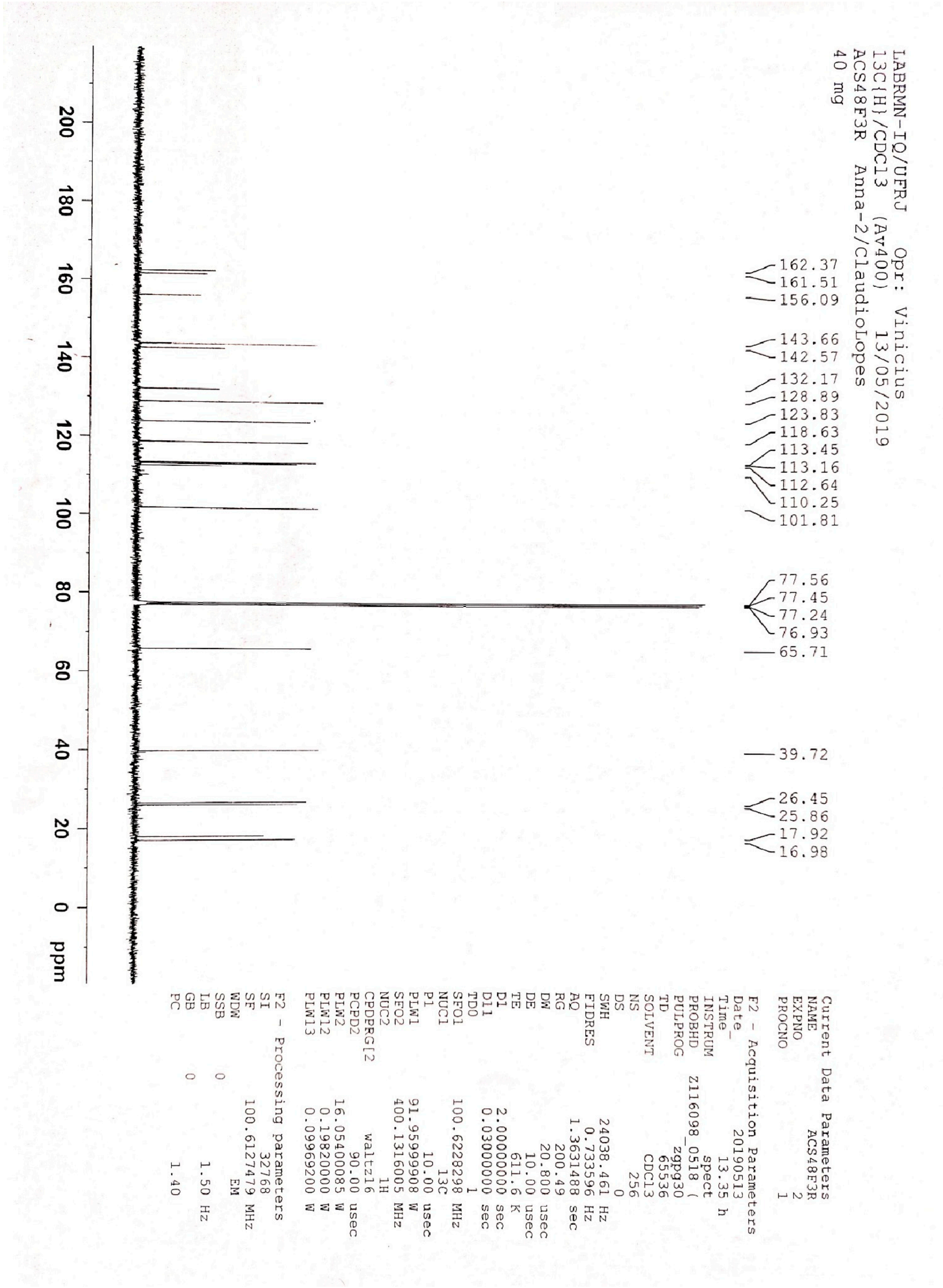

Figure S7. ^{1}H NMR spectra of ACS47.

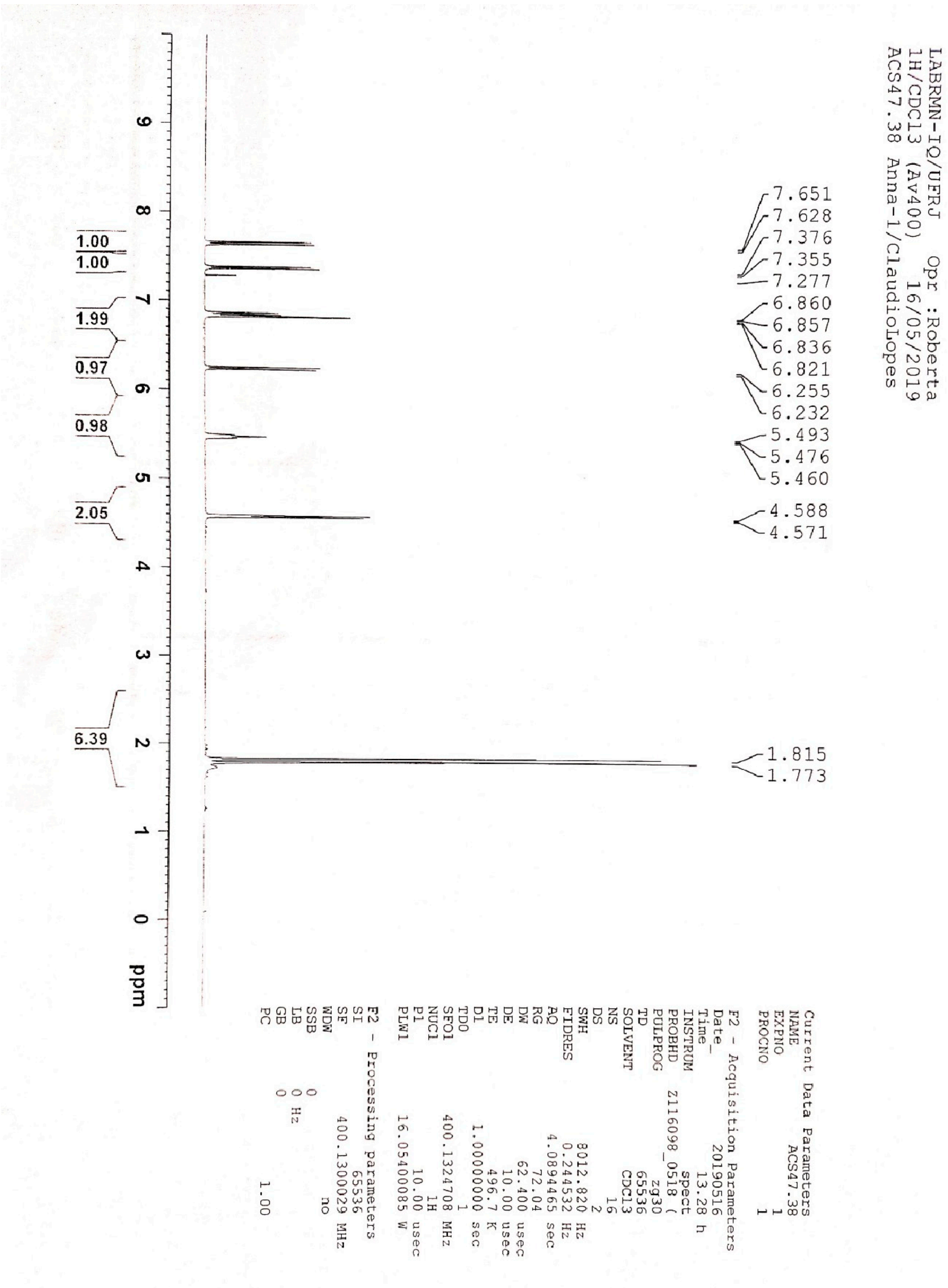

Figure S8. ^{13}C NMR spectra of ACS47.

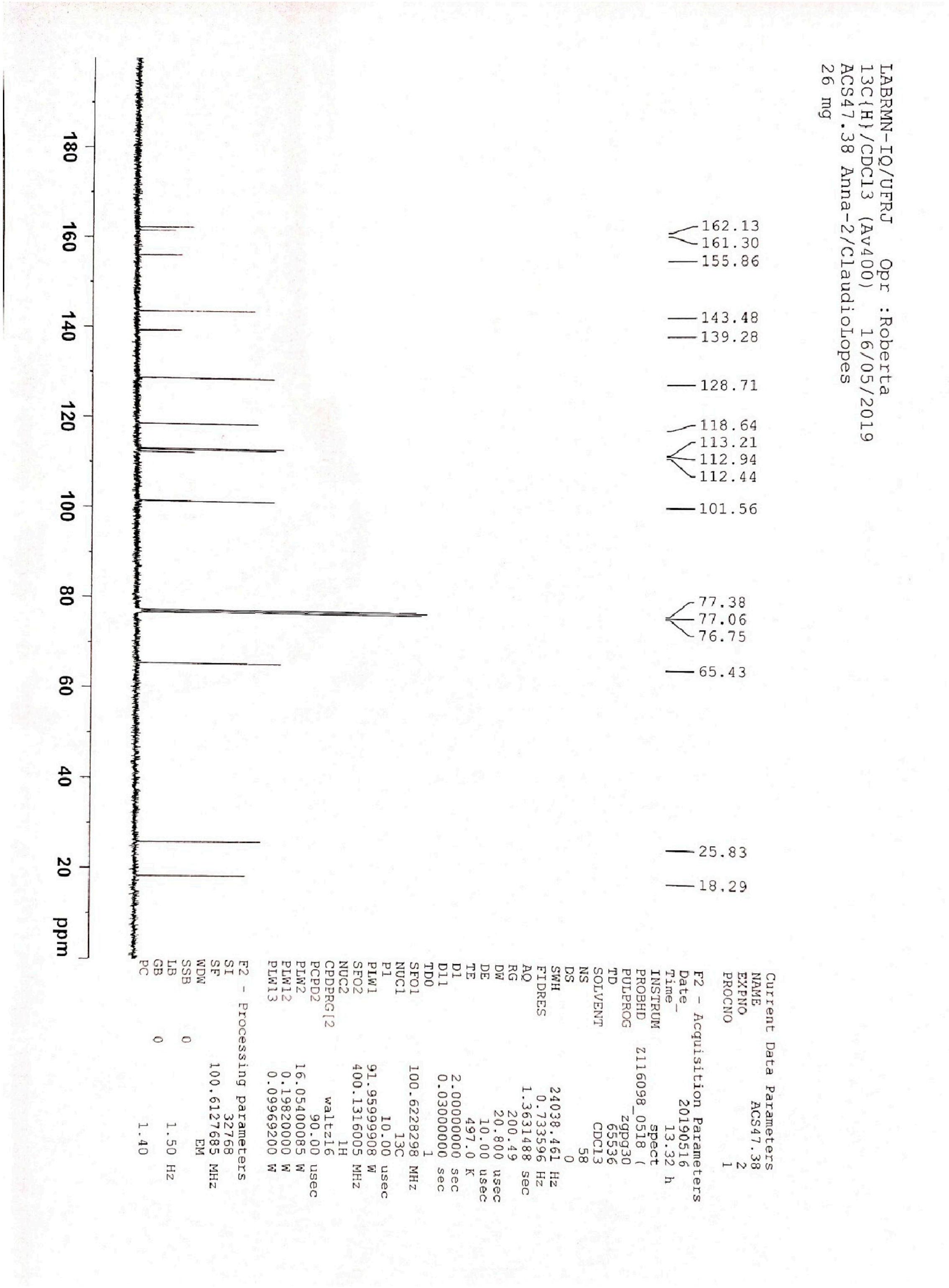

Figure S9. ^{1}H NMR spectra of ACS52.

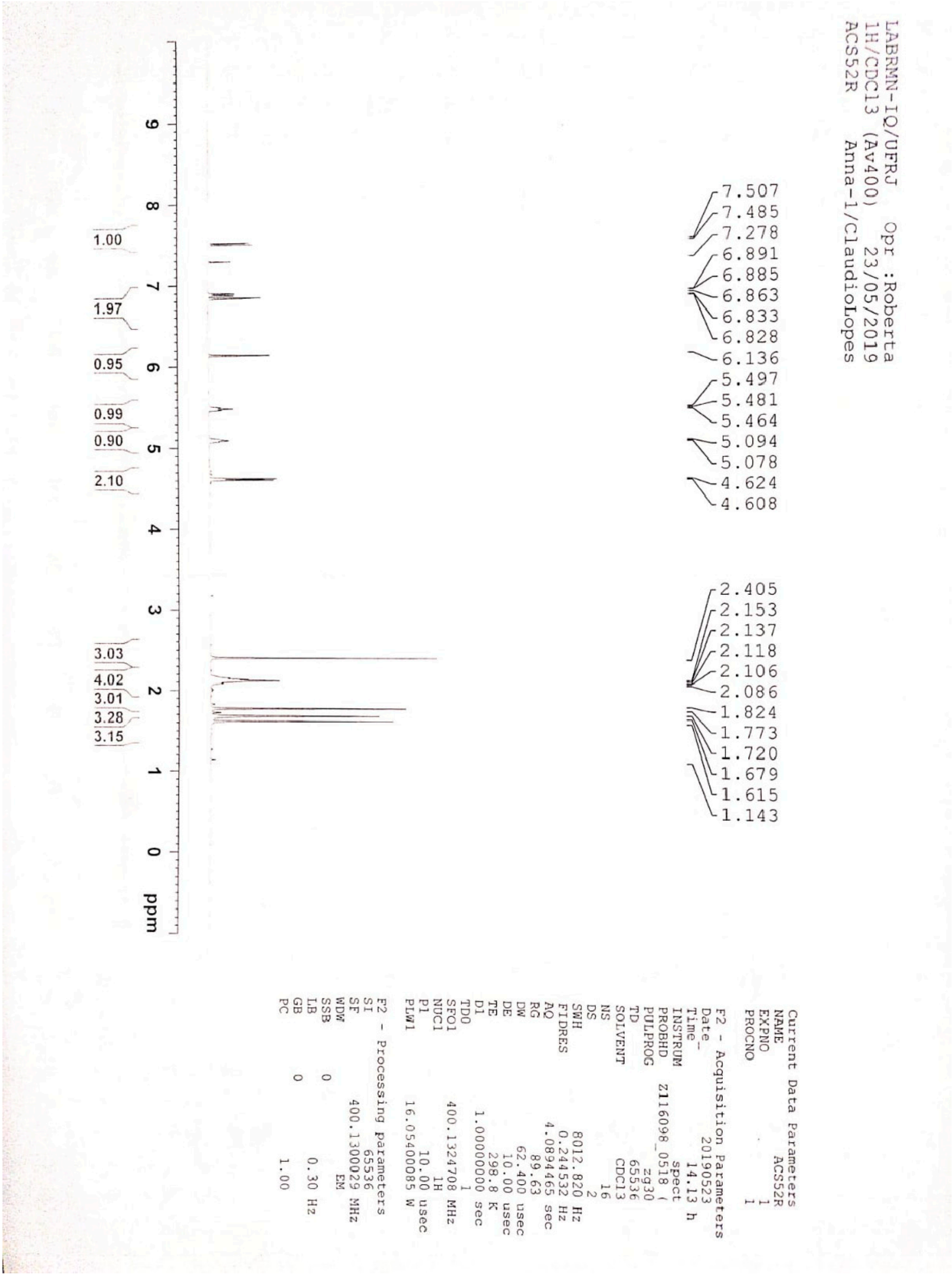

Figure S10. ^{13}C NMR spectra of ACS52.

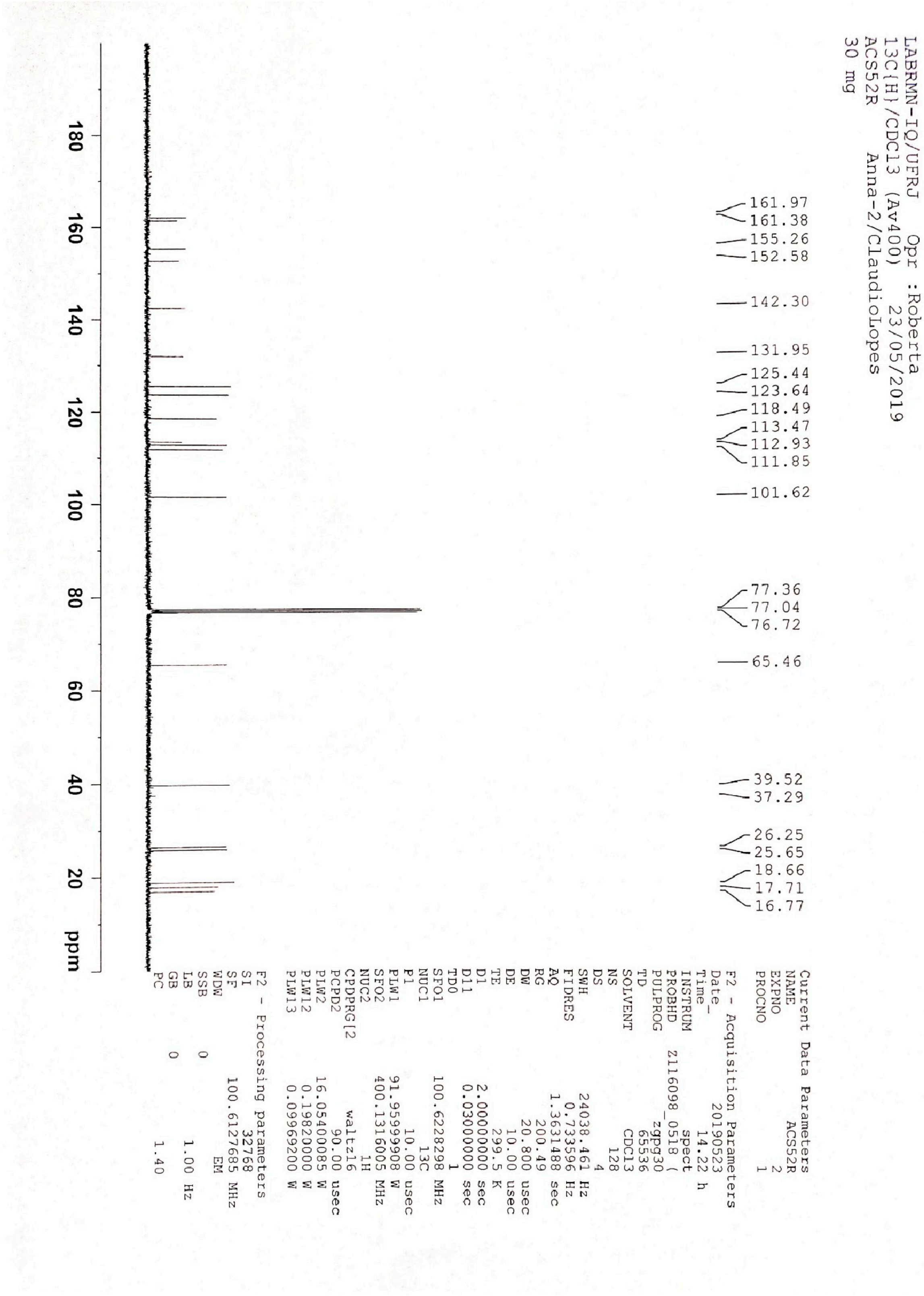

Figure S11. ^{1}H NMR spectra of ACS54.

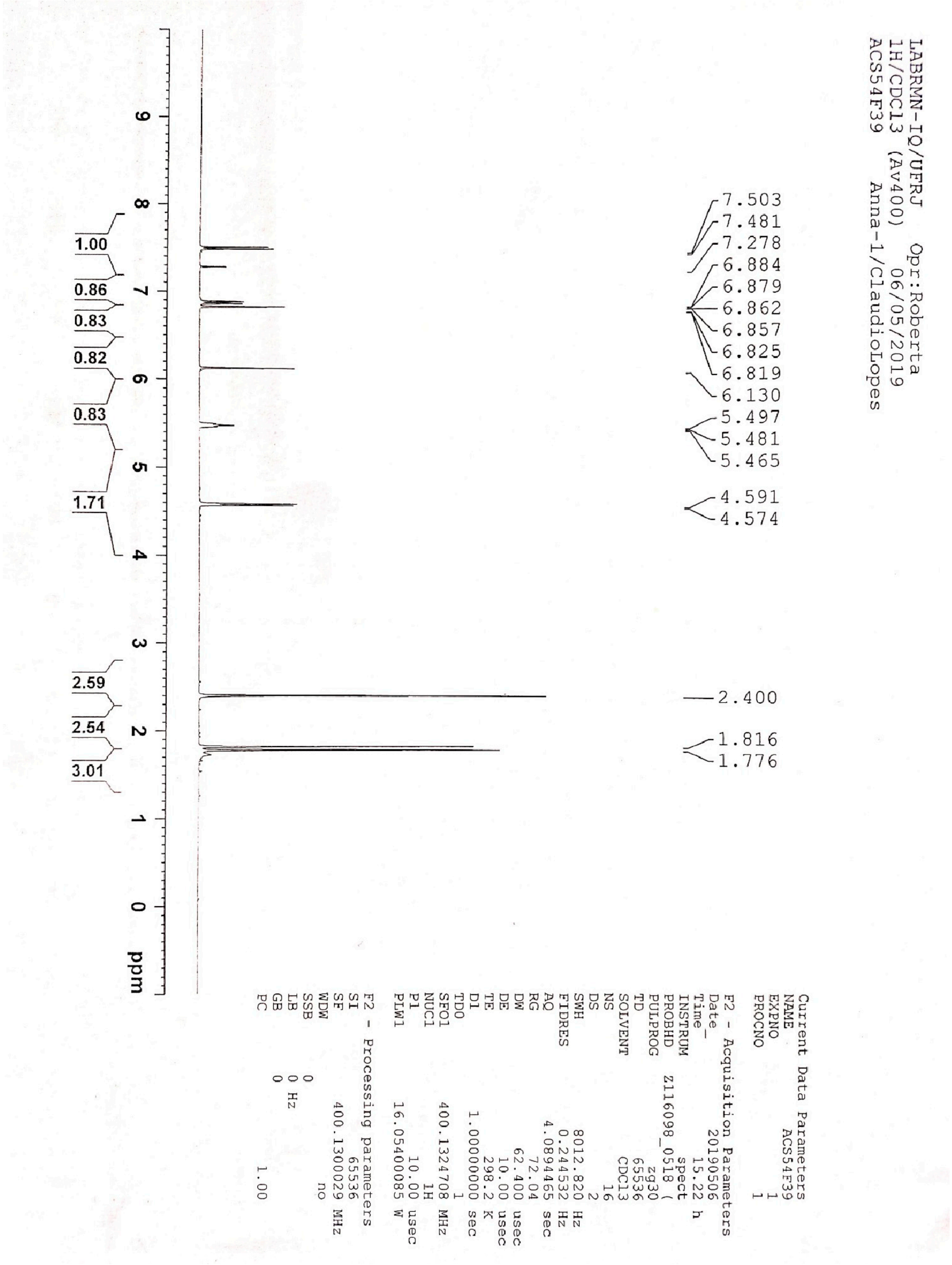

Figure S12. ^{13}C NMR spectra of ACS54.

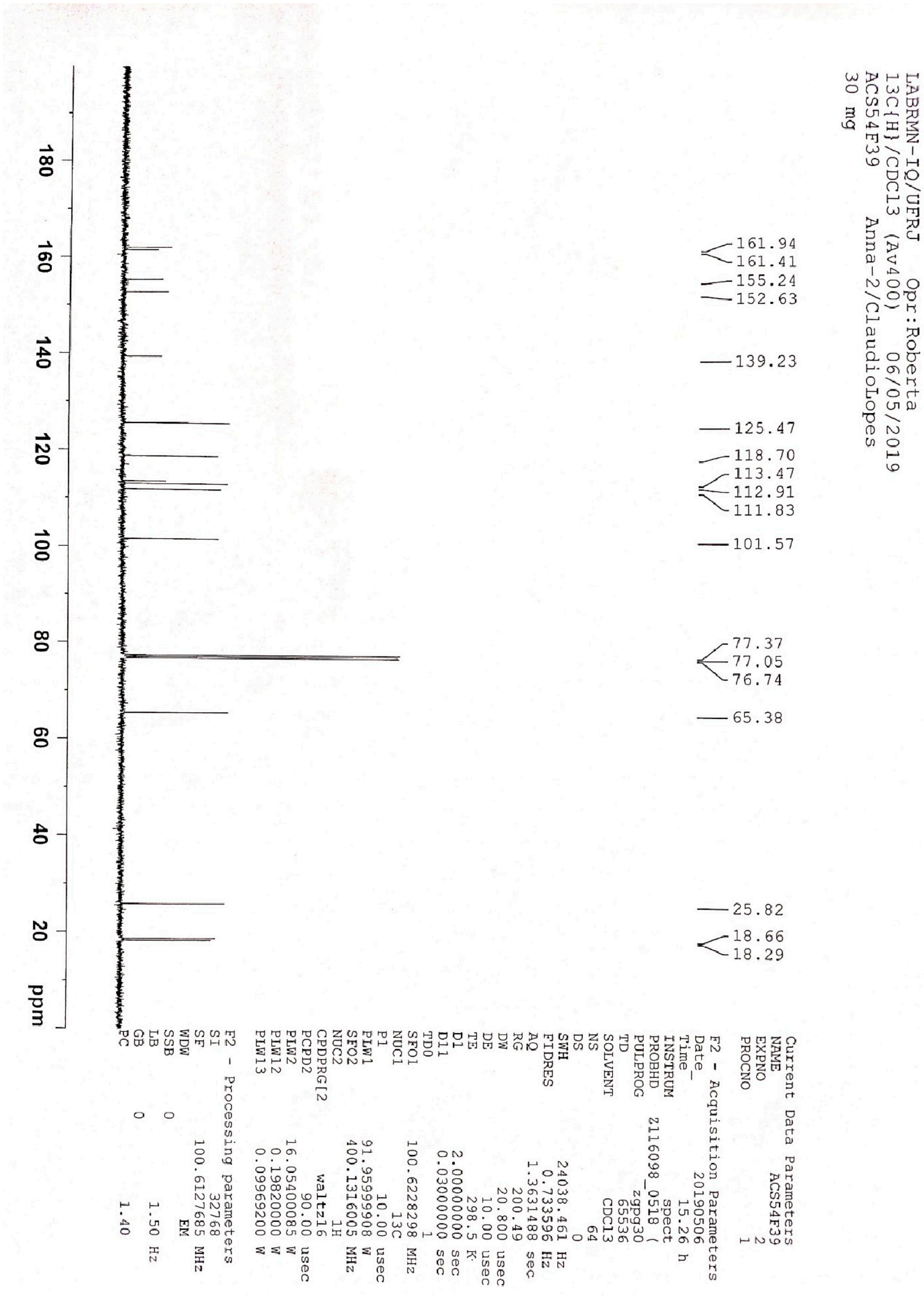

Figure S13. ^{1}H NMR spectra of ACS56.

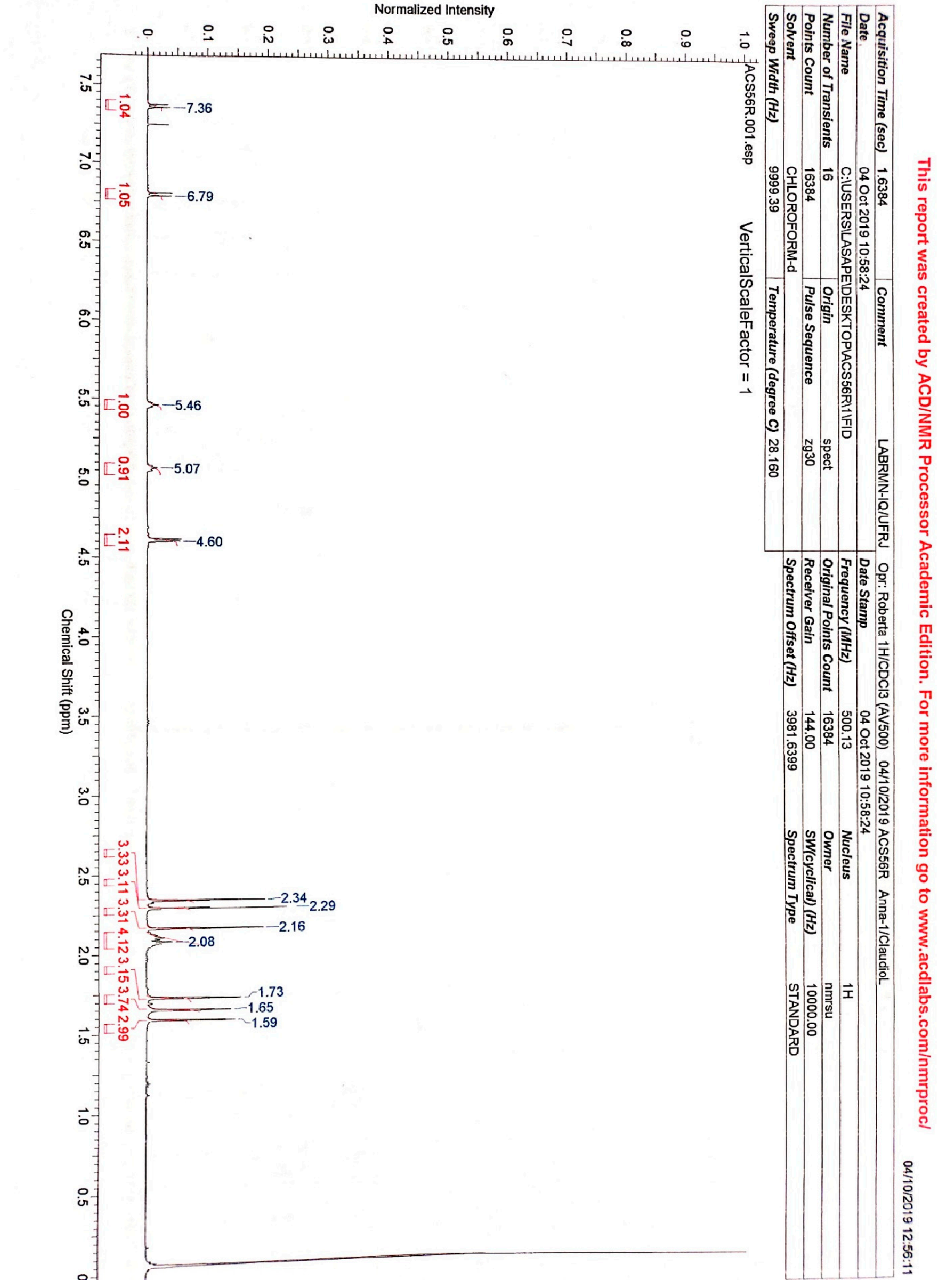

Figure S14. ^{13}C NMR spectra of ACS56.

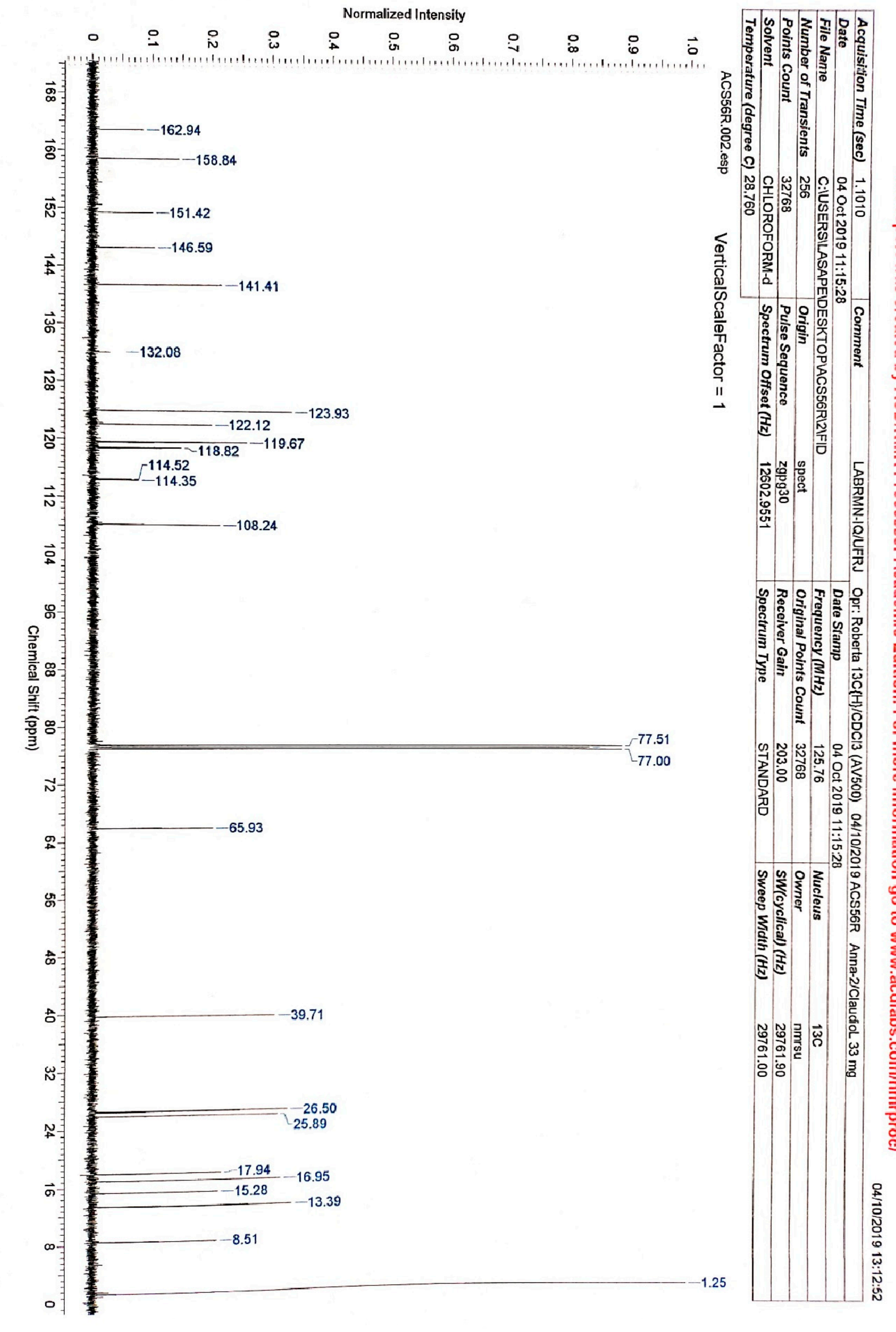

Figure S15. ^{1}H NMR spectra of ACS55.

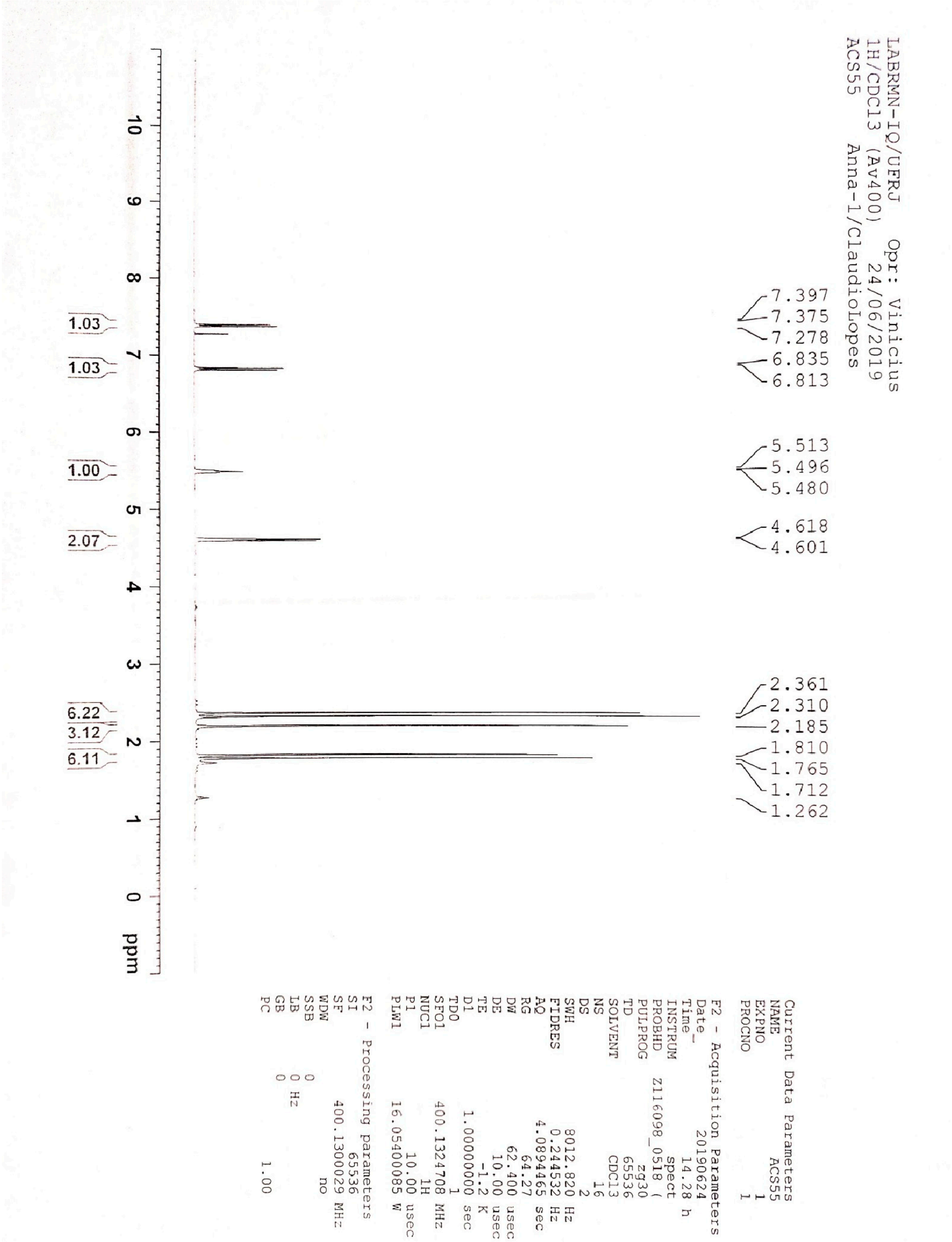

Figure S16. ^{13}C NMR spectra of ACS55.

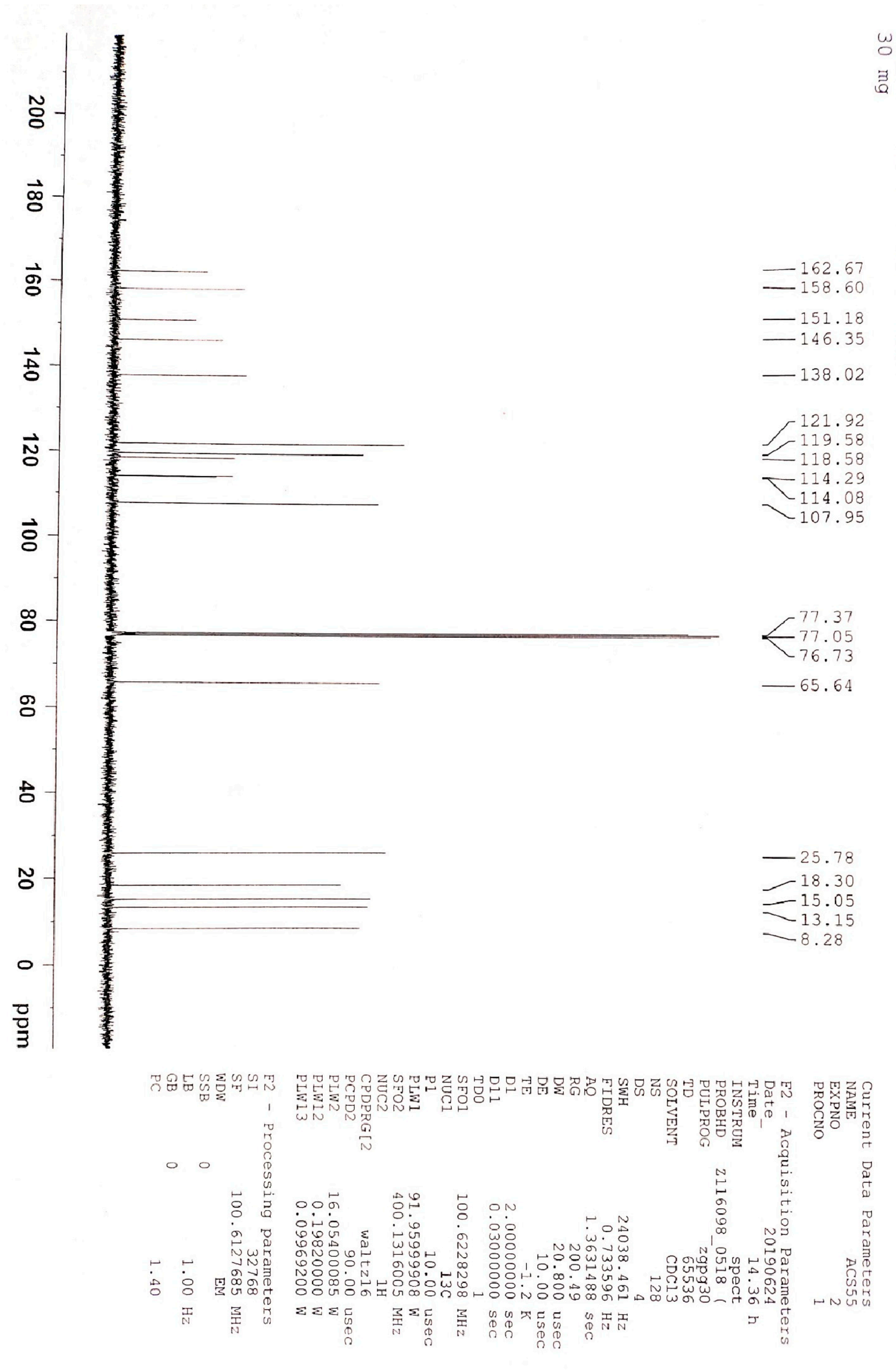

Supplement: Supplementary file 1 [file jof-09-00758-s001.zip › jof-2475213-supplementary.pdf]
